# Stratified Sampling of Neighborhood Sections for Population Estimation: A Case Study of Bo City, Sierra Leone

**DOI:** 10.1371/journal.pone.0132850

**Published:** 2015-07-15

**Authors:** Roger Hillson, Joel D. Alejandre, Kathryn H. Jacobsen, Rashid Ansumana, Alfred S. Bockarie, Umaru Bangura, Joseph M. Lamin, David A. Stenger

**Affiliations:** 1 Information Technology Division, Naval Research Laboratory, Washington, District of Columbia, United States of America; 2 Department of Global and Community Health, George Mason University, Fairfax, Virginia, United States of America; 3 Njala University, Bo, Sierra Leone; 4 Mercy Hospital Research Laboratory, Bo, Sierra Leone; 5 Center for Bio/Molecular Science and Engineering, Naval Research Laboratory, Washington, District of Columbia, United States of America; University of Oxford, VIET NAM

## Abstract

There is a need for better estimators of population size in places that have undergone rapid growth and where collection of census data is difficult. We explored simulated estimates of urban population based on survey data from Bo, Sierra Leone, using two approaches: (1) stratified sampling from across 20 neighborhoods and (2) stratified single-stage cluster sampling of only four randomly-sampled neighborhoods. The stratification variables evaluated were (a) occupants per individual residence, (b) occupants per neighborhood, and (c) residential structures per neighborhood. For method (1), stratification variable (a) yielded the most accurate re-estimate of the current total population. Stratification variable (c), which can be estimated from aerial photography and zoning type verification, and variable (b), which could be ascertained by surveying a limited number of households, increased the accuracy of method (2). Small household-level surveys with appropriate sampling methods can yield reasonably accurate estimations of urban populations.

## Introduction

### Background

The population of a region of interest must be estimated if one’s goal is to convert incidence counts into rates. This conversion is not always necessary, because some epidemiological parameters can now be estimated from incidence counts alone, including the interval between successive cases, and the reproductive number *R*_0_, which is the average number of secondary cases attributable to a primary cause [1, 2]. If these parameters are insufficient to evaluate the models, it may be necessary to calculate the total population *N*. The 5 brief examples that follow illustrate both the necessity of doing so, and some of the difficulties that may be encountered.

In resource-limited environments, it may be possible to use both aerial imagery and limited residential survey data to estimate the population of a region of interest, as shown in the first two examples. Using ground truth data for the measured population of 20 sections in Bo City, Sierra Leone, we compared the uncertainty of estimating the population using survey data for either (1) occupants per residence or (2) rooftop area per resident. The latter variable was computed by manually digitizing the rooftop areas of residential structures in 5 sections of Bo, and calculating the ratio of rooftop area per occupant for each residence [3]. The ability to rapidly estimate the population of both temporary and unplanned settlements is critical for planning resource allocation for refugee and internally displaced populations as well as for places undergoing rapid unplanned urbanization, since in these settings there is usually not a stable residential population. Checchi et al. [4] have developed a two-step method for estimating a refugee population that requires (1) estimating the number of temporary residential structures from satellite imagery and (2) estimating the mean occupancy per structure. The product of the estimate (1) “number of structures” and (2) “mean number of persons per structure” yields an estimate of the total refugee population.

As shown in the next 2 examples, if salient population data are available either directly or by interpolation; derived rates of infection, immunity, or morbidity may be calculated. The standard SEIR (Susceptible, Exposed, Infectious, Recovered) compartmental epidemiological model [5, 6] requires *N* as a parameter. Glasser et al. [6] simulated the implementation of two different influenza vaccination policies, in order to predict their effect on both the incidence of infection and the rate of morbidity. They applied a SEIR model parameterized by demographic parameters for the United States (2005), including the total population stratified by age. The age-specific death rates attributable to pneumonia and influenza were estimated, as were the death rates from all other remaining causes. Gomez-Elipe et al. [7] have developed a model for forecasting the incidence of Malaria in Karuzi, Burundi (1997–2003). To convert the reported instances of malaria to a rate, the investigators divided the rate by the 2006 population census, after rescaling (decrementing) by the population growth factors for the intervals from 1995–2000 (growth factor = 1.32) and 2000–2005 (growth factor = 3.29).

In demographically-diverse environments, different methods may be required to estimate the population at different locations, as shown in our final example. The *GRUMPv1 (Global Rural-Urban Mapping Project, Version One)*, separates the urban population density estimates from the population of the surrounding areas. In addition to enumerated city population data, city footprints can be established by analyzing nighttime satellite images, but this approach may fail to capture small informal settlements in Africa and rural Asia [8] (page 9). Accordingly, several corrections are applied for poorly illuminated settlements [8] (page 9), and point estimates are provided for settlement populations exceeding 1,000. Many models utilize GRUMP for epidemiological modeling, including [8, 9].

### Proposed analysis

In a previous study [3], a *Finite Population Bootstrap* (FPB) [10] (page 92) was used to compare the relative uncertainty of two population estimators: an occupancy-based estimator and a rooftop area-based estimator. For the region of interest, the former was estimated as the product of (1) the average number of persons per residential structure multiplied by (2) the total number of residential structures; and the latter was calculated as (1) the average number of persons per rooftop area (i.e., persons per *m* ^2^) multiplied by (2) the total estimated rooftop area in *m* ^2^. Both estimators were effective, but the uncertainty was about 20% less for the occupancy-based estimator [3] (page 10). Both the occupancy-based and rooftop area-based population estimators were evaluated by simulating *simple random sampling without replacement* (SRSWOR).

The analysis in this current paper will evaluate the use of stratified sampling for population estimation, and will demonstrate the reduction in the uncertainty of the population estimate achievable relative to SRSWOR. Two different stratification designs will be explored: (1) optimal stratification by “persons per structure” and (2) stratified single-stage cluster sampling. The relative advantages and restrictions of both methods will be discussed. The city of Bo itself is approximately 30.1 *km* ^2^ in area, and is divided into 68 uniquely-shaped neighborhoods or *sections* [11](see Fig 1 in [3] and Table 1). These sections vary in size from 0.02 *km* ^2^ (Toubu) to 2.33 *km* ^2^ (Bo Government Reservation). For 20 of the 68 sections, residential survey data are also available [3] (see Table 1). The ground truth survey data for these 20 sections will provide the basis for simulated sampling using different stratification protocols, and for quantifying the reduction in the uncertainty of the population estimate achievable.

**Table 1:** Bo municipal survey data tabulated by section.

(1) Section	(2) Area (*km* ^2^)	(3) Residential Structures	(4) Total Structures	(5) Households	(6) Persons	(7) Mean Number of Persons per Residence
Roma	0.04	4	52	22	139	34.75
Moibawo Farm	0.50	17	43	22	135	7.94
Dodo	0.05	26	88	85	597	22.96
Bo Central	0.07	33	103	51	273	8.27
Toubu	0.02	34	46	88	454	13.35
Kpetewoma	0.20	46	105	94	640	13.91
Komende	0.20	56	258	175	1103	19.70
Salina	0.47	59	231	110	580	9.83
Reservation	2.33	66	252	86	637	9.65
Kindia Town	0.15	102	278	206	1160	11.37
Lewabu	0.48	105	117	170	879	8.37
New York	1.51	116	605	176	1088	9.38
Njai Town	0.22	127	269	388	2298	18.09
New Site south	0.69	136	194	190	1248	9.18
Tengbewabu	0.68	136	233	185	1068	7.85
Yemoh Town	0.40	152	284	289	1858	12.22
Kissi Town	0.20	154	287	400	2490	16.17
Kulanda Town	0.29	197	314	637	3882	19.71
Nduvuibu	0.49	205	343	439	2552	12.45
New London	0.60	208	495	498	2873	13.81
Grand Total	—	1979	4597	4311	25954	—

A summary of the residential and household survey data for 20 municipal sections of Bo (1), showing the area of each section (2); the total number of residential structures, combined residential and non-residential structures, households, and persons per section (3–6); and the mean occupancy per residential structure (7) [3, 11].

The first approach, optimal stratification by persons per structure, requires that the number of persons per structure be already known for all residential structures; possibly from a prior survey or census data. The objective is to exploit this prior data to design an improved stratification protocol for *re-estimating* the population, and to demonstrate a significant reduction in the uncertainty of the population estimate relative to random sampling. Single-stage cluster sampling is useful if the number of sections that can actually be sampled is restricted, perhaps because of cost or schedule limitations. In our examples, the simulated cluster sampling will be restricted to 4 of the 20 available sections. We will investigate the reduction in uncertainty that can be achieved by using a stratified cluster sampling protocol, rather than random selection, to select the 4 sections on each simulation trial. Each section will be completely sampled.

Note that choice of population estimators is independent of the stratified sampling protocol selected for simulated data collection. A stratified Horvitz-Thompson [12] population estimator will be evaluated for all examples. We have also extended our original FPB model to support stratified sampling [10], and partial results from the latter will be contrasted with estimates obtained using the stratified Horvitz-Thompson estimator. Neither the stratified FPB nor the Horvitz-Thompson estimator were used in the prior study.

More specifically, we will address the following 4 questions:

1. What reduction in the uncertainty of the population estimate can be achieved by stratified sampling—relative to simple random sampling of all sections—if the residential survey records are first partitioned into mutually-exclusive strata with non-overlapping ranges of “persons per residential structure?”
2. Can any reduction in uncertainty be achieved—again relative to simple random sampling of all sections—if the *sections* are partitioned into mutually-exclusive and exhaustive strata, rather than partitioning the individual records (PSUs) into strata?
3. For single-stage cluster sampling, if the sections (clusters) are partitioned into mutually-exclusive strata by “total residential structures per section,” what is the relative reduction in uncertainty that can achieved using stratified cluster sampling, rather than unclassified cluster sampling?
4. Does stratification by the “total persons per section”—if known—further reduce the uncertainty of the single-stage cluster population estimates?

We will use a single dataset developed previously in [3] (see Table 1). This dataset contains individual records for each of 1,979 residential structures surveyed. Each record includes the number of persons in the structure, a variable that we will utilize in this paper. The survey methodology and data collection methods used to construct the dataset analyzed in this manuscript were all developed previously. The original articles [3, 11] should be consulted for a complete discussion. The current article complements and extends these prior studies, but does not supplant them.

The utility of these methods for the 5 initial examples, which were presented to establish the importance of estimating the population of a region of interest, will depend upon the availability of partial survey data for occupancy, the existence of adequate estimates of the total number of residential structures, and the presence of stable patterns of residential occupation. Neither method is likely to be useful for improved estimation or re-estimation of the population of a highly transient population living in temporary shelters as described by Checchi et al. [4].

### Model development

The simulations described in this investigation were written in the programming language R [13]. Supporting functions from multiple R libraries were used, including [14–16]. Additional custom code was written and tested by the first author. The R package *stratification* [16, 17] provides algorithms for finding the optimal boundaries for a variable *Y*, based on criteria proposed by Lavallée and Hidiroglou [18]. This package supports several different heuristics, including Kozak’s algorithm [19, 20] which can also find the optimized boundaries for a specified sample size *n*.

In all of the examples presented here, the true optimal boundaries were found through exhaustive search. Given the relatively small size of the dataset (1,979 records), all possible combination of strata boundaries were tested to determine which set minimized the uncertainty of the population estimate as a function of sample size [17] (page 33).

## Methods

### Survey methodology and dataset development

#### Ethics Statement

All data collection involving human subjects was approved by a total of three independent Human Subjects Research Institutional Review Boards: Njala University, George Mason University, and the U.S. Naval Research Laboratory. Written informed consent was obtained from each household representative who participated in the survey. Survey data were obtained as part of a broader study to determine not only population demographics but health metrics and health care utilization trends.

#### Terminology

Structures in Bo City were divided into two categories. “Nonresidential” structures included governmental, commercial, and nonprofit organizational structures such as places of worship. “Residential” structures included all structures used as sleeping quarters. Fig 1 in [3] shows the 20 sections in which the surveys were conducted. Some surveyors were staff of Mercy Hospital Research Laboratory (MHRL); most were Master of Public Health students at Njala University. The surveyors received several days of training, including instruction on geographic data collection using hand-held GPS units, interviewing techniques, and research ethics—including an emphasis on confidentiality. During the interviews, one representative—an adult of either sex—served as a representative of each household. Each residential record lists the number of persons reported living within the same residential structure, and the number of separate households. No attempt was made to differentiate between persons based on gender, age, or household affiliation.

#### Protection of human subjects

This field work was a joint task of Njala University, George Mason University, and the U.S. Naval Research Laboratory. Institutional review boards (IRB) at all three institutions approved the data collection methodology.

#### Bo City dataset

Our sampling frame is a list of 1,979 residential structures encompassing 20 of the 68 sections in Bo City. For each residential structure, there is a unique single record listing the number of persons and households; because these records can be randomly selected, this database will provide the basis for simulated sampling of residential structures. By definition, each residential structure is also a Primary Sampling Unit (PSU). A cluster is defined as a logical collection of PSUs [21](page 24); in this study, a cluster and a Bo City section will be treated as synonymous in the context of single-stage cluster sampling.

### Overview of stratified sampling

**Fig 1:**
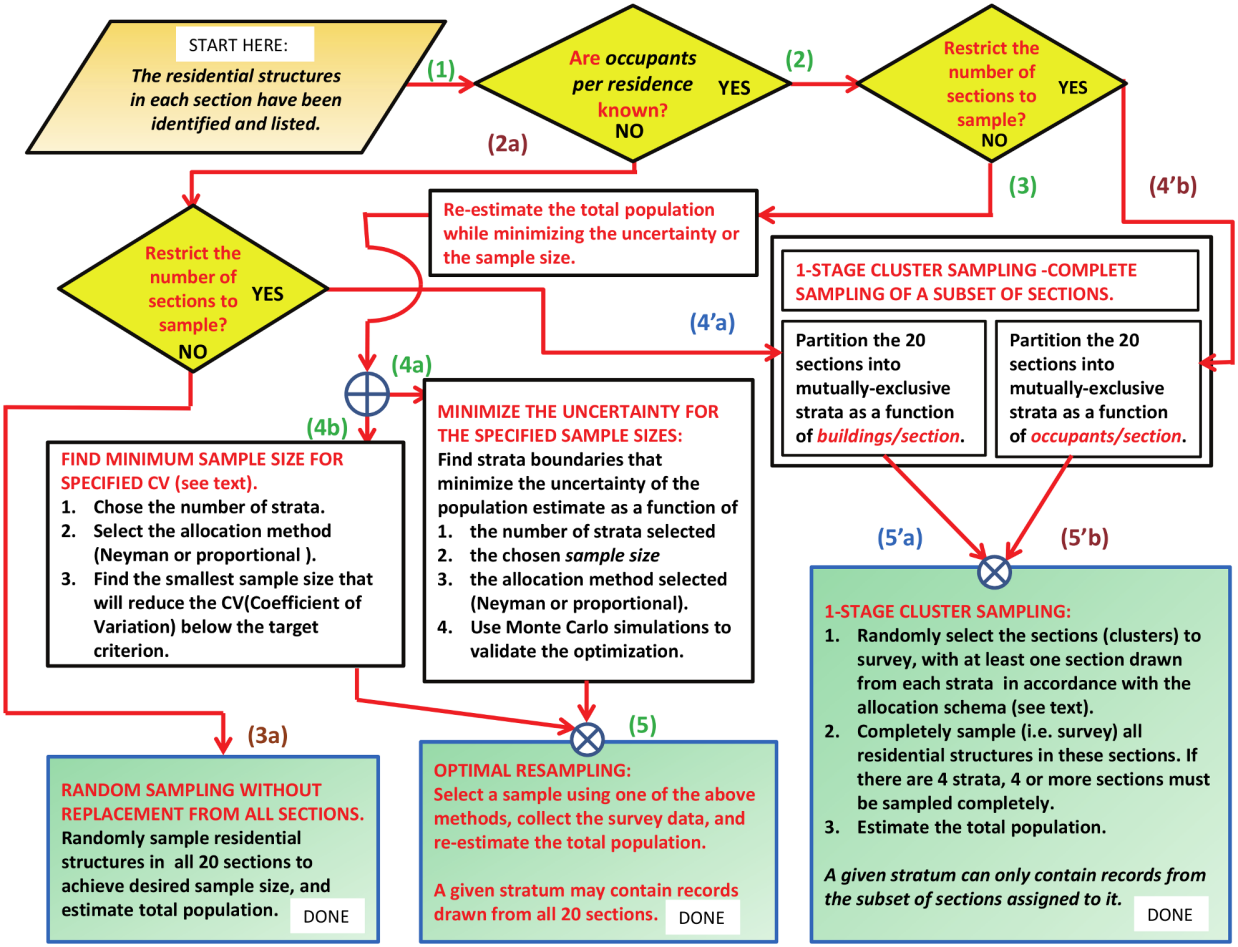
Flow chart for stratified sampling protocols. This figure summarizes all of the optimization and control protocols for stratified sampling developed in this study. See text for a summary of each major protocol and its corresponding steps through the flow chart. The light brown parallelogram is the starting point for all protocols, the yellow diamonds are decision boxes, and the light green squares denote the process end states.

The flowchart in Fig 1 summarizes the algorithms and simulations that will be developed in the text. The objective of this study is to investigate alternative approaches for stratified sampling of the residential structures in a resource-limited environment, and to determine the relative reduction in the uncertainty of the estimate of the total population—if any—that results. In all cases, it is assumed that at least the number of residential structures in each section are known. This flowchart may be referenced as the two major protocols are developed and simulated in detail.

#### Optimal stratification by persons per residence

As with any stratified sampling scheme, the PSUs (Primary Sampling Units) —the 1,979 individual residential structures (see Table 1)—must first be divided into mutually-exclusive and exhaustive strata [21] (page 121). After the stratification boundaries have been determined, simulated sampling can be executed. Based on pilot studies, we determined that 4 levels of stratification would be sufficient for proof of concept. The stratification and estimation algorithms will be summarized later. The survey variable *X* and the stratification variable *Y* are the same—specifically, the number of persons per residential structure. For this reason, it was not necessary to model the relationship between *Y*, the measured survey variable (persons per residential structure), and *X*, the stratification variable [17].

On each simulation trial, a subset of the PSUs were randomly selected from each stratum as a function of (1) the total sample size and (2) the allocation algorithm selected. This step created a stratified sample of the PSUs. A stratified Horvitz-Thompson estimator was then used to re-estimate the total population of the 20 pooled sections [12, 17, 21]. Referring to Fig 1, the objective was to use the previously collected survey data to design a survey protocol that would:

1. reduce the uncertainty of the estimated population as a function of sample size relative to random sampling without replacement:
  (1) → (2) → (3) → (4*a*) → (5).
2. and/or find the minimum sample size needed to minimize the *Coefficient of Variation (CV)* below some specified threshold:
  (1) → (2) → (3) → (4*b*) → (5)

#### Stratified single-stage cluster sampling

When schedule or resources restrict the survey to a subset of sections within the region of interest, single-stage cluster sampling can be applied. (If there is no restriction on the number of sections to be sampled, all sections can be sampled without replacement for a given sample size.) Assume that the number of residential structures per section is known, but not the number of persons per section. The 20 sections will first be partitioned into the desired number of mutually-exclusive strata, using the section sizes (i.e., total residential structures per section) as the stratification variable; see Table 1 for these values. Each residence in a section will be assigned to the same stratum. For each trial of the stratified single-stage clustering protocol, one section will be selected from each stratum, and all of the residences in the selected sections will be completely sampled. For the control case, the same number of sections will be selected, but the stratification boundaries will be ignored. In effect, in the control case, all sections will be assigned to a single stratum.

In Fig 1:

(1) → (2*a*) → (4′*a*) → (5′*a*)

If the total population of each cluster is known, “total persons per section” can be used as the stratification variable, rather than “total residential structures per section.” The relative uncertainty of the population estimate for single-stage cluster sampling will be further reduced.

(1) → (2) → (4′*b*) → (5′*b*)

Single-stage cluster sampling may also be executed without stratification, but in the simulations that follow, the uncertainty of the population estimate will be roughly doubled for the unstratified case. The cluster sampling protocol is appropriate when financial or schedule constraints impose limits on the number of sections to be sampled. The advantages of stratified cluster sampling are:

1. No auxiliary data is required other than a count of residential structures in each of the 20 sections under consideration. If the total population of each section is available, an even more efficient design can be realized.
2. A cluster design permits a trade-off between the size of the survey, the number of sections sampled, and the uncertainty of the population estimate.

#### Optimal stratification

Let *L* strata be defined on the stratification variable *X*, the number of persons per residential structure. Number the strata *h* = 1, 2…, *L*. Define the boundaries of the strata as *b*_*h* = 1_, *b*_*h* = 2_, …, *b*_*h* = *L*_. Stratum *h* will include all values of *X* in the interval [*b*_*h*−1_, *b*_*h*_) such that *b*_*h*−1_ < *X* ≤ *b*_*h*_.

Assume that there is a total of *N* units or records that are being stratified. An optimal solution of the values *b*_1_ ≤ *b*_2_ ≤ *b*_*L*−1_ for a sample of size *n* minimizes the following objective function [19] (Eq 3):

$$n=n(b_{1},b_{2},...,b_{L-1})=$$ (1)

$$N_{L}+{\left(\sum_{h=1}^{L-1}W_{h}S_{h}\right)}^{2}{\left({\bar{Y}}^{2}c^{2}+1/N\sum_{h=1}^{L-1}W_{h}S_{h}^{2}\right)}^{-1}$$ (2)

where

1. *N*_*L*_ is the size of stratum *h*
2. *W*_*h*_ = *N*_*h*_/*N* is the proportion of the total units (records) in *N* assigned to stratum *h*
3. *S*_*h*_ is the standard deviation of the stratification variable *Y* in stratum *h*
4. $\bar{Y}$ is the population mean of the survey variable *Y*
5. *c* is the CV (coefficient of variation) of the survey variable *Y*
6. *N* is the total number of records or units being partitioned into strata

#### Allocation selection

When the strata boundaries are optimized for a given sample size *n*, the coefficient of variation of *Y* is minimized [17]. Note that the constraint for optimization is dependent not only on the distribution of the stratification variable *Y*, but also upon the allocation rule used. The allocation rule chosen will determine the weights *W*_*h*_. The allocation rule used in the R package *stratification* [16] is developed in [22].

Let *a*_*h*_ be the proportion of samples assigned to the *h* ^*th*^ stratum. Then:

$$\sum_{h=1}^{L-1}a_{h}=1$$ (3)

Given a total sample size *n*, the sample sizes for each “take-some” stratum will be:

$$n_{h}=\left(n-N_{H}\right)a_{h}$$ (4)

where

$$a_{h}=\gamma_{h}\sum_{h=1}^{H-1}\gamma_{h}$$ (5)

and

$$\gamma_{h}=N_{h}^{2q1}{\bar{Y}}_{h}^{2q2}var{\left[Y\right]}_{h}^{2q3}\left(p_{i}=1,2,3\right)$$ (6)

Setting *q*1, *q*2 and *q*3 to (0.5, 0.0, 0.5) parameterizes Neyman’s allocation for each stratum, while (0.5, 0.0, 0.0) corresponds to proportional allocation. When Neyman’s allocation is used, a sample size *n*_*h*_ may be equal to or greater than the number of available PSU’s *N*_*h*_. The stratum may then be categorized as a “take-all” stratum [17], and every record (i.e. PSU) in the stratum will be selected, rather than a subset of the stratum records. If necessary, the sample sizes of one or more of the remaining strata are transparently incremented to realize the desired total sample size *n*.

Neyman allocation will minimize the variance (i.e. uncertainty) of the stratified population estimator. The Neyman allocation for a sample of size *n* is equivalent to the expression below [21] (page 158):

$$n_{h}=\{\frac{N_{h}\sigma {}_{h}y}{\sum_{h=1}^{L}N_{h}\sigma_{hy}}\}\left(n\right)$$ (7)

#### The Horvitz-Thompson estimator

The Horvitz-Thompson (H-T) estimator provides an unbiased estimate of the total population from either a stratified or unstratified sample, provided the inclusion probabilities are greater than zero for each unit sampled [23]. Let the sample size be *n*, the value of the *i* ^*th*^ individual record or unit be *y*_*i*_, and define *π*_*i*_ = *n*_*h*_/*N*_*h*_ as the inclusion probability for the *i* ^*th*^ record in strata *h*. (For the important special case where all units are assigned to a single stratum, *π*_*i*_ = *n*/*N*, and all units are assigned the same probability of inclusion. In the text, this is referred to as the control case.) For simple random sampling without replacement, the Horvitz-Thompson estimator is then:

$${\hat{Y}}_{\pi }=\sum_{h=1}^{L}\sum_{i\in n_{h}}\frac{y_{i}}{\pi_{i}}$$ (8)

This expression could be simplified, but the double summation makes clear that the total population estimate is the sum of the weighted estimates for the individual strata.

#### Optimal stratification for resampling

In our first set of demonstrations, we evaluated a design for resampling a known population for which complete survey data exists [19]. Using the optimization approach described earlier, the 1,979 units were divided into 4 strata, using the number of persons per residential structure as the sampling variate *Y*. The choice of *L* = 4 as a reasonable number of strata was based on the findings from preliminary simulation studies. Five different random sample sizes were selected: 330, 660, 990, 1,320, and 1,650 records, out of the total 1,979 records available. Simulations were run using both proportional and Neyman allocation.

For each sample size, 1,000 random trials were run. In each trial, a stratified sample was selected, and the Horvitz-Thompson population estimate calculated. The inclusion probability *π*_*h*_ for each record in the sample was calculated as shown in Table 2.

**Table 2:** Neyman-optimized allocation as a function of sample size and stratum.

A					
	*nh*[*h*] = sample per size per stratum[h]				
sample size	*nh*[1]	*nh*[2]	*nh*[3]	*nh*[4]	∑(*nh*)
330	75	67	72	116	330
660	102	103	110	345	660
990	126	134	94	636	990
1320	143	138	128	911	1320
1650	114	1	1	1534	1650
B					
	*Nh*[*h*] = total houses/stratum[h]				
sample size	*Nh*[1]	*Nh*[2]	*Nh*[3]	*Nh*[4]	∑(*Nh*)
330	694	649	439	197	1979
660	569	611	454	345	1979
990	445	520	378	636	1979
1320	314	380	374	911	1979
1650	180	134	131	1534	1979
C					
	π_*h*_ = *h*[*h*]/*Nh*[*h*]				
	sample size	*h* = 1	*h* = 2	*h* = 3	*h* = 4
	330	0.11	0.10	0.16	0.59
	660	0.18	0.17	0.24	1.00
	990	0.28	0.26	0.25	1.00
	1320	0.46	0.36	0.34	1.00
	1650	0.63	0.01	0.01	1.00
D					
	upper boundary limits (persons per residence)				
	sample size	*h* = 1	*h* = 2	*h* = 3	*h* = 4
	330	8.50	14.50	24.50	86.00
	660	7.50	12.50	19.50	86.00
	990	6.50	10.50	14.50	86.00
	1320	5.50	8.50	11.50	86.00
	1650	4.50	5.50	6.50	86.00

Table 2a: Optimal samples per stratum as a function of sample size. Table 2b: Optimal allocation of residential structures per stratum as a function of sample size. Table 2c: The inclusion probability π_*h*_ = *h*[*h*]/*Nh*[*h*] as a function of sample size. Table 2d: The upper strata boundaries as a function of sample size.

Table 2a lists the number of residential structures to be sampled in each stratum for optimal stratification of the variable “persons per residential structure.” Table 2b is the total number of residential structures per stratum, while Table 2c specifies the ratios of samples per stratum divided by the total number of residential structures per stratum. These ratios are not constant for each sample size because the optimization was constrained by Neyman allocation, rather than proportional allocation. Table 2d lists the upper boundary limits as a function of sample size.

#### Stratified finite population bootstrap

The samples drawn for each stratum were also concatenated and resampled [10, page 97], [14, 24] creating a bootstrap sample of size *n*_*h*_ for each strata. The *n*_*h*_ samples from each strata were then combined to create a single sample of size *n* (330, 660, 990, …), and the total population was estimated using the FPB. For the control group and the proportional allocation case, the estimated population obtained using the FPB was compared with the results from the Horvitz-Thompson estimations. (Neyman allocation could not be compared, since the individual bootstrap estimates for each stratum required proportional allocation.)

The FPB model mirrored the decrease in uncertainty observed with the H-T estimator using optimal proportional allocation, but the variance of the FPB is greater. The average ratio of the 0.95 confidence intervals between the H-T estimator and FPB estimator was approximately 0.70 for the control group, and 0.58 when comparing the estimators for optimal proportional allocation. A paired t-test was used to compare the intervals, and *P* < 0.001 in both cases. For the control case, 67% of the H-T estimators fell within the 0.50 confidence interval for the FPB, quantifying the greater uncertainty of the FPB estimator. Likewise, comparing the proportionally-allocated 4 strata case, 76% of the H-T estimators fell within the 0.50 confidence interval for the FPB. The FPB used is one of a family of finite population bootstrap algorithms. A recent study [24] compared the variance characteristics of different implementations of the FPB, and proposed a new FPB algorithm may present reduced uncertainty relative to the implementation used here.

#### Relative uncertainty of the population estimates

**Fig 2:**
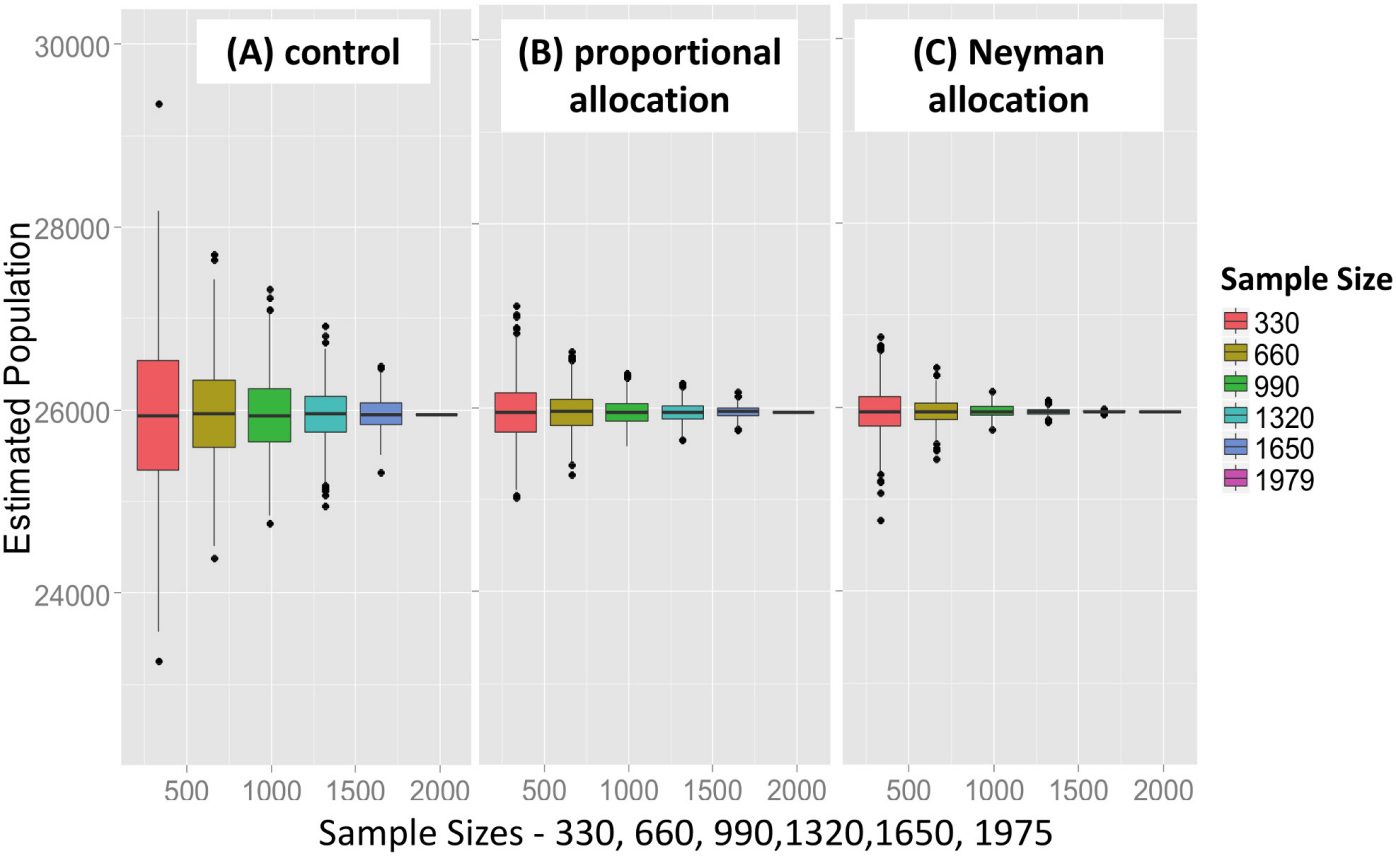
Relative uncertainty of optimized Horvitz-Thompson population estimates. Quantile boxplots (0.25, 0.75) showing the distribution of the stratified Horvitz-Thompson population estimates as a function of sample size and stratification protocol. The bar in each box is the median value of the estimate, while outliers deviating by one or more quantiles from the median are denoted as discrete points. (A) control—all 20 sections are placed in a single stratum (B) 4 strata, with proportional allocation for sample selection (C) 4 strata, with Neyman allocation for sample selection. Persons per residence was used as the stratification variable, and there were 1,000 simulations for each boxplot.

Fig 2 illustrates the [0.25, 0.75] quantile boxplots as a function of sample size for the H-T estimator for the single-stratum control case (A), and using proportional (B) and Neyman (C) allocation, respectively. The mean ratios of the 0.95 confidence intervals were 0.58 and 0.19, respectively (*P* < 0.001 and *P* < .005). In summary, the uncertainty using optimal stratification with Neyman allocation was roughly 20% of the uncertainty observed for the single stratum control group, averaged over 1,000 simulations.

#### Coefficient of Variation optimization

A single example will be provided for optimizing the Coefficient of Variation, as illustrated schematically in Fig 1. The *CV* is equal to the *RRMSE*, the Relative Root Mean Squared Error. The target value of the *CV* was set to be ≤ 0.01. When 4 levels of stratification were requested, Neyman allocation was enabled, and “persons per residential structure” was selected as the stratification variable. The critical minimal sample size returned by the stratification algorithm was 456. The upper stratification boundaries (persons per residential structure) were:

1. *stratum 1:* 9.5
2. *stratum 2:* 17.5
3. *stratum 3:* 31.5
4. *stratum 4:* 86.0

#### Single-stage cluster sampling

Table 3 shows the results of applying the Neyman stratification algorithm. For a sample of some specified number of clusters (sections), the recommended number of sections to select are given for each stratum. The variable *bh*[*h*] specifies the upper boundary in “residential structures per section” for each stratum *h*. The stratification algorithm actually returns the first three boundaries, since the upper boundary of the 4th stratum is the maximum possible value of the stratification variable, which is 208—the number of residential structures in the New London section. The variable *nh*[*h*] indicates the allocated number of clusters that should be selected from each stratum for a balanced sample of a given size in clusters (sections). Given 4 stratification levels, the minimum number of clusters that can be selected is 4, and the recommended sample allocation is (1, 1, 1, 1). A comparable table was generated for proportional allocation, and for an allocation of (1, 1, 1, 1) sections per stratum, the stratification partition was identical. Table 4 shows the stratification by section for the 4-section allocation (1, 1, 1, 1), which was used in the simulations, and the 10-section allocation (2, 1, 6, 1) provided for comparison.

**Table 3:** Optimal cluster allocation as a function of sample size.

(1) Number of Clusters Selected	(2) Minimum Number of Records Per Sample	(3) *nh*[*h*] = sample size in clusters (sections) for stratum = *h*				(4) *Nh*[*h*] = total number of clusters (sections) for stratum = *h*				(5) *bh*[*h*] = upper boundary for stratum[*h*].			
–	–	h = 1	h = 2	h = 3	h = 4	h = 1	h = 2	h = 3	h = 4	h = 1	h = 2	h = 3	h = 4
4	373	1	1	1	1	6	5	6	3	51.0	110.5	175.5	208.0
5	454	1	1	2	1	5	4	8	3	40.0	84.0	175.5	208.0
6	570	1	1	3	1	5	4	8	3	40.0	84.0	175.5	208.0
7	697	1	1	4	1	5	4	8	3	40.0	84.0	175.5	208.0
8	714	2	1	4	1	5	4	8	3	40.0	84.0	175.5	208.0
9	850	2	1	5	1	5	4	8	3	40.0	84.0	175.5	208.0
10	986	2	1	6	1	5	4	8	3	40.0	84.0	175.5	208.0
11	1138	2	1	7	1	5	4	8	3	40.0	84.0	175.5	208.0
12	1164	3	1	7	1	5	4	8	3	40.0	84.0	175.5	208.0
13	1780	1	1	1	12	2	3	3	12	21.5	40.0	62.5	208.0
14	1839	1	1	1	13	2	3	2	13	21.5	40.0	57.5	208.0
15	1883	1	1	1	14	2	2	2	14	21.5	33.5	51.0	208.0
16	1895	1	1	13	2	2	3	13	2	21.5	40.0	201.0	208.0
17	1929	1	1	6	10	2	2	6	10	21.5	33.5	103.5	208.0
18	1886	2	2	2	12	2	3	3	12	21.5	40.0	62.5	208.0
19	1946	2	1	6	10	2	2	6	10	21.5	33.5	103.5	208.0
20	1979	2	2	2	14	2	2	2	14	21.5	33.5	51.0	208.0

Stratification variables for selecting clusters (sections) for one-stage stratified cluster sampling with 4 levels of stratification (*L* = 4). The entries in each column are (1) the number of clusters to be selected (2) the minimum number of PSU’s (i.e. residential structures) spanned by the selected clusters if the allocation *nh*[*h*] is drawn (3) the number of clusters (sections) *nh*[*h*] to be drawn from each stratum (4) the total number of clusters *Nh*[*h*] in each stratum and (5) the upper boundary *bh*[*h*] in units of “residential structures per cluster” for each of the four strata. A comparable table was constructed for stratification by population per section, but is not shown for the sake of brevity.

**Table 4:** Neyman stratification of Bo sections by “residential structures per section” and “persons per section.”

			Stratification variable: “residential structures per section”		Stratification variable: “persons per section”	
(1) Section	(2) Residential structures per section	(3) Persons per section	(4) allocation = (1, 1, 1, 1)	(5) allocation = (2, 1, 6, 1)	(6) allocation = (1, 1, 1, 1)	(7) allocation = (2, 1, 6, 1)
Roma	4	139	1	1	1	1
Moibawo Farm	17	135	1	1	1	1
Dodo	26	597	1	1	1	2
Bo Central	33	273	1	1	1	1
Toubu	34	454	1	1	1	2
Kpetewoma	46	640	1	2	1	2
Komende	56	1103	2	2	2	3
Salina	59	580	2	2	1	2
Reservation	66	637	2	2	1	2
Kindia Town	102	1160	2	3	2	3
Lewabu	105	879	2	3	2	3
New York	116	1088	3	3	2	3
Njai Town	127	2298	3	3	3	4
New Site south	136	1248	3	3	2	4
Tengbewabu	136	1068	3	3	2	3
Yemoh Town	152	1858	3	3	3	4
Kissi Town	154	2490	3	3	3	4
Kulanda Town	197	3882	4	4	4	4
Nduvuibu	205	2552	4	4	3	4
New London	208	2873	4	4	4	4

4-level Neyman stratification boundaries for the cluster (section) list stratified by the *number of residential structures* per section (cols. 4–5), and the *number of persons* per section (cols. 6–7). In practice, the latter may be unknown. An allocation of (1, 1, 1, 1) sections per stratum is optimal for a sample size of 4 sections, which was used in our examples. The (2, 1, 6, 1) allocation, shown for comparison, is optimal for a 10-section sample. See Table 3.

In our implementation of simulated single-stage cluster sampling, the allocation used on each trial was (1, 1, 1, 1), because the objective was to estimate the population while minimizing the number of sections sampled. A single section was selected from each stratum, as discussed previously. In the control protocol, 4 sections were selected randomly without replacement from the unconstrained population of 20 sections. The form of the Horvitz-Thompson for single-stage cluster sampling is [21] (page 336):

$$\;{\hat{Y}}_{HTE}=\sum_{i=1}^{v}\sum_{i\in n_{h}}\frac{y_{i}}{\pi_{i}}\;$$ (9)

1. *y*_*i*_ = the total number of persons for *i* ^th^ cluster (section)
2. *π*_*i*_ = the probability of the *i* ^th^ cluster being sampled during this trial
3. *v* = the total number of clusters sampled (i.e., 20)

This estimator provides an unbiased estimate of the total population.

## Results

### Optimal stratified sampling

The relative efficiency of optimal stratification by persons per residential structure has already been discussed. As shown in Fig 2, the uncertainty of the population estimation with Neyman allocation was roughly 20% of the uncertainty observed for the unstratified control group, averaged over 1,000 simulations. Table 5 compares the variance and standard error of the mean (SEM) of the Horvitz-Thompson estimators for 1,000 simulated single-stage cluster sampling trials, selecting a fixed sample size of 990 records.

$$SEM=\sigma /\sqrt{(}n)*FinitePopulationCorrection$$ (10)

$$=\sigma /\sqrt{(}n)*\sqrt{(}\left(N-n\right)/\left(N-1\right))$$ (11)

$$=\sigma /\sqrt{(}990)*\sqrt{(}\left(1979-990\right)/\left(1978\right))$$ (12)

**Table 5:** A comparison of uncertainty for unstratified, proportional-, and Neyman-allocated population estimates.

(1) Optimal re-estimation of total population (1000 simulation trials)	(2) Number of residential structures per sample	(3) Mean value of H-T estimator for 1000 trials	(4) Standard deviation of the H-T estimator	(5) Variance of the H-T estimator	(6) Standard Error of the Mean (SEM)
(A) Unstratified	990	25942	412.18	169892.35	9.26
(B) Proportional allocation	990	25950	142.23	20229.37	3.20
(C) Neyman allocation	990	25956	71.53	5116.54	1.61

A comparison of the variance *σ* ^2^ and the SEM (*Standard Error of the Mean*) of the Horvitz-Thompson (H-T) estimator for 1,000 simulated sampling trials, and a fixed sample size of 990. For the unstratified control case (A), all sections were assigned to a single stratum, in contrast to 4-level optimal stratification using either proportional (B) or Neyman allocation (C). The stratification variable is “persons per residential structure” and Table 2, subtable 2a, specifies the samples per stratum.

The Levene test [25, 26]was used to compare the variances of the stratified protocols with the variance of the unstratified control group. The paired comparisons were blocked by sample size. The null hypothesis for the Levene test is that the ratio of 2 specified variances is equal to 1.0. For all tests, σ_*x*|*N* = *n*_^2^ was the variance for 1,000 simulated trials for sample size of *n* (e.g., 330, 660, 990 …) using 4-level Neyman or proportional allocation, and σ_*c*|*N* = *n*_^2^ the variance 1,000 simulated trials for the comparable unstratified control case. The differences between the variances were statistically significant, with *p* < 0.001 for all comparisons, and the hypothesis that the ratio σ_*x*|*N* = *n*_^2^/σ_*c*|*N* = *n*_^2^ = 1.0 was rejected for all tests.

### Single-stage cluster sampling

**Fig 3:**
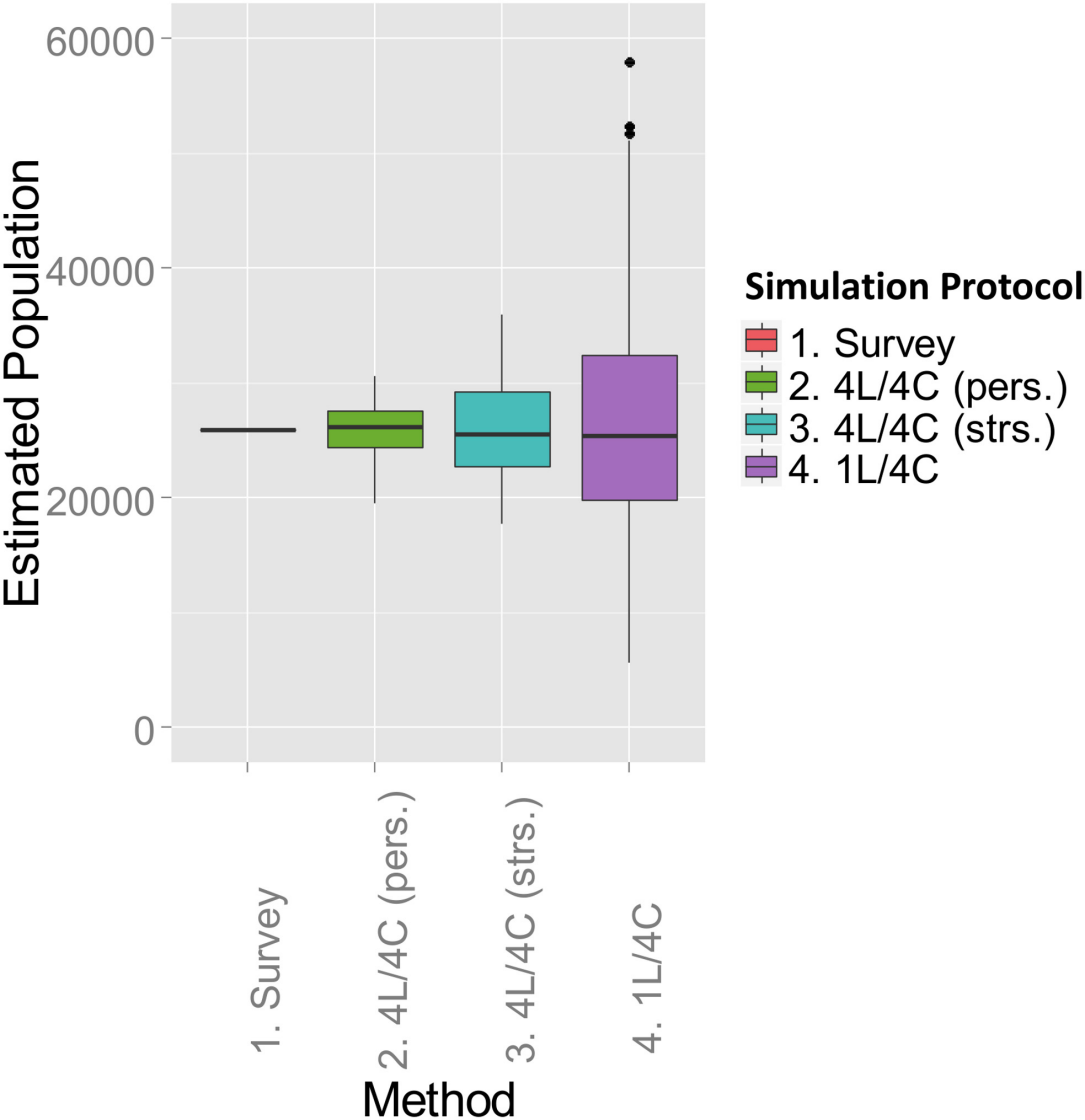
Single-stage cluster sampling. Quantile boxplots for 1,000 stratified 4-level simulated single-stage cluster sampling trials using H-T estimation. The bar in each box is the median value of the estimate, while outliers deviating by one or more quantiles from the median are denoted as discrete points. Four selected sections are completely sampled on each simulation trial. (1) “Survey” is the measured value of the population of the 20 sections (25,954 persons). (2) 4*L*/4*C* (pers.)—4 cluster sample, sections stratified by “persons per section.” (3) 4*L*/4*C* (strs.)—4 cluster sample, sections stratified by “residential structures per section.” (4) 1*L*/4*C*—4 clusters selected at random from the 20 available sections.

Fig 3 shows the box histograms for the single-stage cluster sampling simulations. The uncertainty of the population estimation using stratified cluster selection is about 48% of the uncertainty of the estimation based on random cluster selection, as measured by comparing the [0.25, 0.75] quantile intervals. This difference is significant at *P* < 0.001 (paired t-test).

In single-stage sampling, if a section is selected from one of the four strata, all residences in the section are then included in the sample. Each stratum contains a mutually-exclusive subset of the 20 sections, with non-overlapping ranges of buildings per section between the strata. Because a single section is selected from each stratum for each one-stage survey sample, the sample allocation is balanced with respect to the stratification variable “residential structures per section.” See Table 4, column 4.

Table 6 compares of the variance and standard error of the mean (SEM) of the Horvitz-Thompson estimator for 1,000 simulated sampling trials, selecting 4 sections on each trial. The average number of residences selected per trial are shown in the table. For the unstratified control case, all sections were assigned to a single stratum, in contrast to 4-level optimal stratification using either proportional or Neyman allocation. The variance ratios were again compared between all three protocols using the Levene test. The differences between the variances were statistically significant, with *p* < 0.001 for all comparisons.

**Table 6:** A comparison of simulation results for single-stage cluster sampling.

(1) 1-Stage Cluster Method-1000 trials	(2) Average residences sampled per simulation trial	(3) Mean value of H-T estimator for 1000 trials	(4) Variance of H-T estimator for 1000 trials	(5) Standard Error of the Mean (SEM)	(6) Number of sections sampled
(A) Unstratified	396	26,270	84,413,782	256	4
(B) Stratify by number of buildings per section	443	25,935	16,983,502	115	4
(C) Stratify by number of persons per section	506	25,909	5,503,716	65	4

Comparison of the standard deviation *σ*, the variance *σ* ^2^, and the SEM (*Standard Error of the Mean*) for the single-stage sampling protocol. The uncertainty of the Horvitz-Thompson population estimate decreases as a function of the protocol used to partition the 20 sections of Bo City into 4 strata: (A) unstratified single-stage sampling (B) stratification by number of buildings per section (C) stratification by the total number of persons per section. There are 1,979 residential structures in the 20 sections, and a measured population of 25,954 persons.

If the 4 sections for the single-stage protocol are chosen at random, rather than in accordance with the stratification partition, the uncertainty of the population estimate for 1,000 simulation trials is roughly doubled (Fig 3). This occurs because the sampling protocol is no longer balanced with respect to the number of buildings per section. The probability of selecting a single section from each of the 4 strata is now 11%, rather than 100% (Eq 13). Conversely, almost 90% of the samples drawn will consist of sections drawn from 3 strata or fewer. The theoretical probability that a section will be selected from each of the 4 sections on a given trial is:

$$P_{n=4}\left(strata=1\wedge 2\wedge 3\wedge 4\right)=$$ (13)

$$=(6\times 5\times 6\times 3)/\left(\begin{array}{c} 20\\ 4 \end{array}\right)$$ (14)

=540/4845 (15)

=0.1115 (16)

The above calculation is consistent with the simulation results, in which 119 balanced 4-strata samples were drawn in 1,000 random trials. A comparable argument applies to the simulations using the number of persons per section as the stratification variable.

### Stratification by section for non-cluster sampling

If “persons per structure” are known, optimal stratification boundaries and allocations can be found [18]. Each stratum will contain residences from one or more sections. In single-stage cluster analysis, the sections are partitioned into strata by either “(a) residential structures per section” or “(b) total persons per section,” and one or more sections are selected on each trial from each stratum for complete sampling. As a third possibility, if “persons per structure” are unknown, we may ask whether either of the stratification variables (a) or (b) could be used to efficiently partition the 20 sections into mutually-exclusive strata for *non*-cluster sampling. All of the residences in a given section would be assigned to the same stratum, and a given stratum would contain all of the residential records from the subset of sections assigned to it. A sample of residential records would be drawn from each stratum on a given trial, usually without completely sampling any one section. This protocol could prove advantageous if the proposed partitioning is more efficient than simple random sampling without replacement, even if it is less efficient than optimal stratification by “persons per structure.”

**Fig 4:**
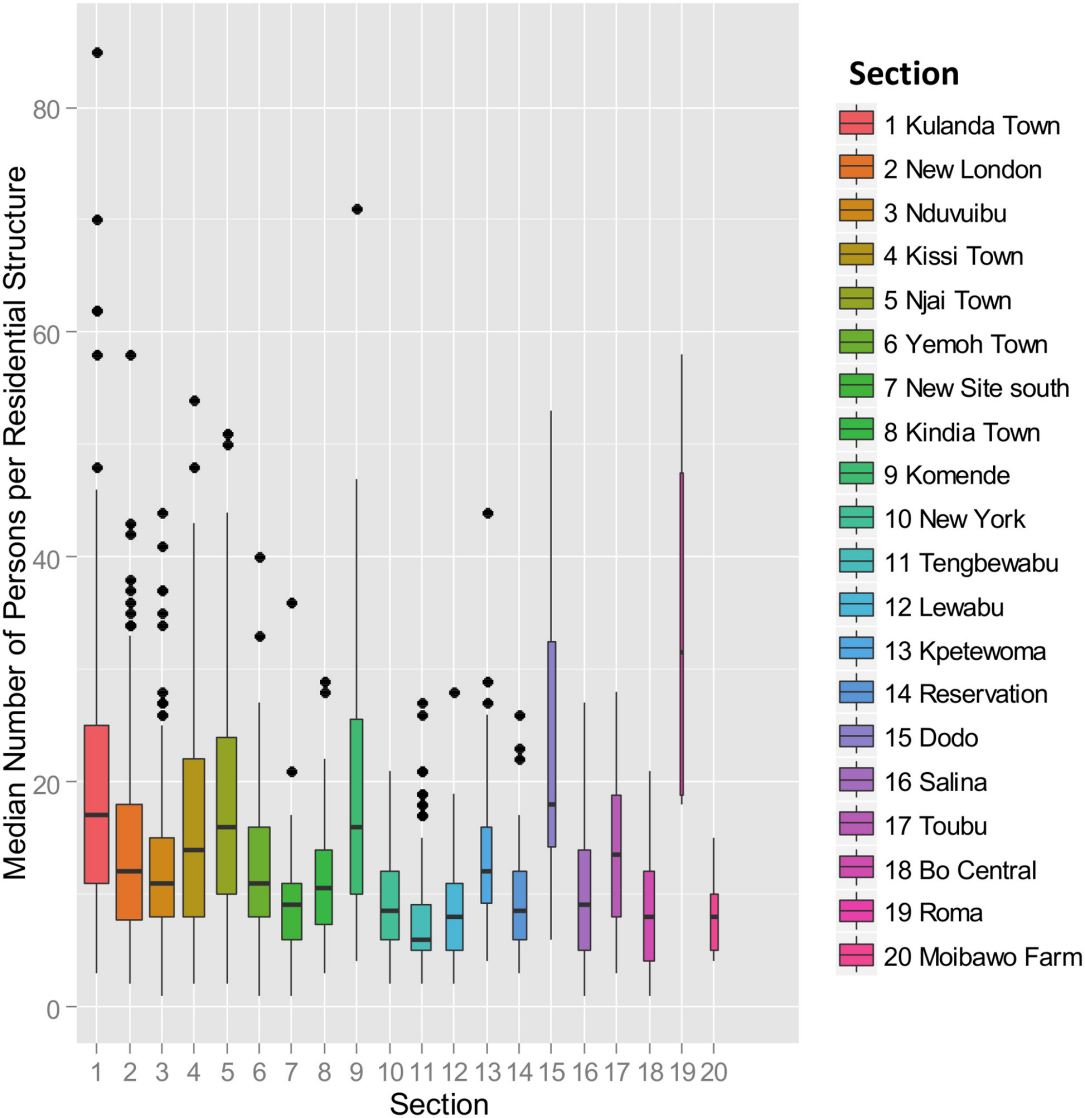
Quantile boxplots for each of the 20 sections. For each section, a quantile boxplot (0.25, 0.75) shows the distribution of the number of persons per residence, arranged in descending order of total section population. The bar in each box is the median value, while outliers deviating by one or more quantiles from the median are denoted as discrete points. The width of each box is proportional to the square root of the number of residential structures (i.e., records) in the section. Roma is an anomaly with 4 residential structures, and 139 total persons.

There are two difficulties with attempting to stratify the data at the section level, rather than at the level of the individual record. For any stratification plan to be viable, the units within a stratum must be relatively coherent with respect to the stratification variable selected. If the stratification variable is “persons per section,” this goal will be difficult to achieve. Fig 4 shows the quantile boxplots for the number of buildings per section, arranged from left to right in order of decreasing number of persons per section. The upper and lower “hinges” correspond to the first and third quartiles (the 25th and 75th percentiles), and the band inside the box is the 2nd quartile (i.e., the median) value of the number of persons per residential structure. The width of each box is proportional to the square root of the number of residential structures (i.e., records) in the section [27]. Roma appears to be anomalous because, although there are only 4 residential structures in this section, there are a total of 139 persons, because these structures are apartment complexes, rather than individual homes. As can be seen, there will be significant overlap between the the ranges of persons per structure for virtually any partitioning of the 20 sections used.

To clarify the above discussion, two experimental simulations were run. The same 4-level partition used for the single-stage cluster sampling was used to define a non-clustered random sampling protocol. Every record in a section was then assigned to the *same* designated stratum. For example, all records for Kulanda Town, Nduvuibu, and New London were assigned to stratum 4—see Table 4. Residences were then randomly selected from all 4 strata, and the number of residences selected from each stratum was proportional to the total number of residences the stratum contains. 1,000 simulated sampling trials were run, using the same sequence of 5 sample sizes used for the optimal stratification analysis (see Table 2). Because each stratum contained records from multiple sections, each sample typically contained records from multiple sections. Conversely, none of the sections were completely sampled on a given trial, in contrast to the protocol for the single-stage cluster model. For a second simulation, the stratification variable “persons per section” was used, rather than “residential structures per section.” See columns 4 and 5 in Table 4. The results are summarized in the next paragraph, but are not presented in a table or figure.

Relative to simple random sampling without replacement of all strata, which was also simulated as a control, the reduction in uncertainty for section-based non-cluster stratification was minimal and statistically insignificant. Levene’s test was again used to compare the ratio of the *σ* ^2^s. The ratio of σ_*x*_^2^/σ_*c*_^2^, where *x* denotes the stratification variable, and *c* denotes the unstratified control case, was 0.95 for stratification by “total persons per section,” and 0.98 for stratification by “residential structures per section,” averaged over the 5 sample sizes. For either stratification method, the hypothesis that the *σ* ^2^ were the same for the 1,000 trial comparisons of the stratified and unstratified population estimates could not be rejected for *p* < 0.05 for any of the 5 sample sizes.

This approach failed to reduce the uncertainty of the estimate because:

1. By design, all residences with a stratum were subsampled, rather than selecting a single section from each stratum to achieve balanced sampling across strata, as was done using a single-stage cluster sampling protocol.
2. There will be considerable overlap in the variable “persons per residential structure” for any possible partition (see Fig 4), although the *range* of section sizes (i.e., number of residences per section) for each stratum was distinct in the constructed example.

**Fig 5:**
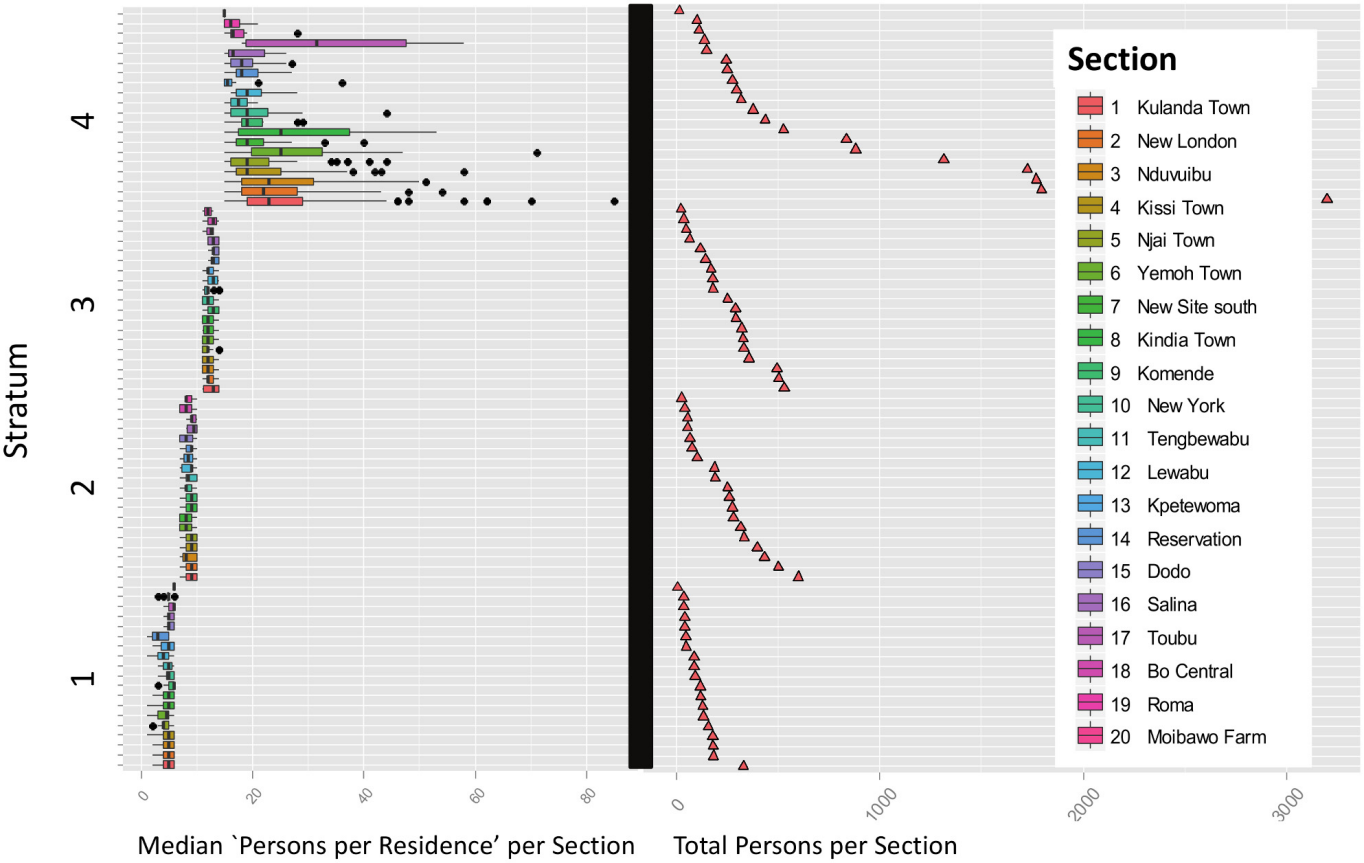
Quantile boxplots for optimal 4-level stratification by “persons per residence.” The 4-level stratification variable is “persons per residence” (Table 2-d). The quantile boxplots [0.25, 0.75] show the partitioning of the records by stratum for all 1,979 records. The bar in each box is the median value of persons per residence, while outliers deviating by one or more quantiles from the median are denoted as discrete points. The samples in a given stratum may be assigned from any of the 20 eligible sections. The optimized Neyman allocation has completely separated the 4 strata with respect to overlapping values of the stratification variable.

**Fig 6:**
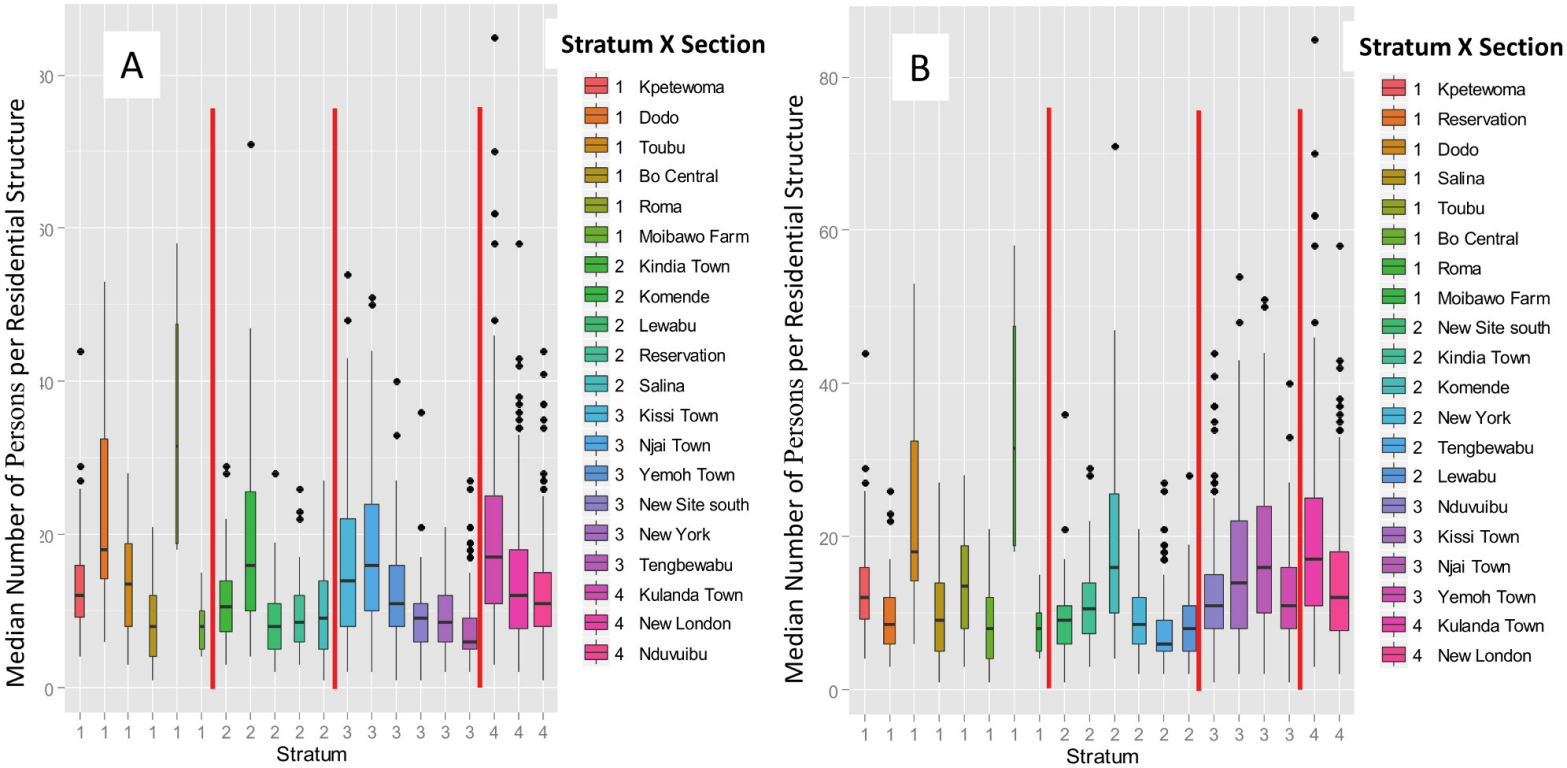
Quantile boxplots for single-stage cluster stratification by (A) “residential structures per section” and (B) “total persons per section”. (A) For the single-stage cluster sampling, the 20 sections were partitioned into 4 proportionally-allocated stratification levels. Within each stratum, the sections are arranged in descending order of total persons. The stratification variable is the total number of residential buildings per section (see Table 4). The quantile boxplots show the partitioning by stratum of the 1,979 records in the database, although only a subset of 4 sections will be drawn on a single simulation trial. The bar in each box is the median value of “persons per residence,” while outliers deviating by one or more quantiles from the median are denoted as discrete points. (B) Quantile boxplots showing stratification by total persons per section. This stratification approach requires that the population of each section be known, in contrast to stratification by residential structures per section.

In this context, it is also instructive to compare Figs 5 and 6. Fig 5 shows the distribution of the unit records (i.e., persons per residence) as a function of the stratification boundaries for a Neyman allocation for a sample of size 990. See Table 2. All 1,979 records are shown in the box histograms. In each stratum, the records can be selected from any of the 20 eligible sections. Note that there is complete separation between the 4 stratum-specific distributions of the stratification variable “persons per residence.” In contrast, Fig 6 shows the comparable distributions of the unit records as a function of the 4-level stratification by residential structures per section (A) and persons per section (B) to support single-stage cluster sampling. In both cases, the records within a section are assigned to a *single* stratum, which results in considerable overlap between the number of persons per residence within the same stratum. Although there is an apparent grouping, the coherence within the strata is relatively weak, and the strata are not well separated, as in Fig 5. Stratification by “persons per section” is relatively efficient for single-stage cluster sampling because a single section will be completely sampled from each stratum, and the ranges of residential structures per section are non-overlapping between strata.

The second difficulty is operational, and not specific to this dataset. The stratification boundaries were determined as a function of the number of residential structures per section. But all sections contain both residential and non-residential structures, as shown in Table 1. If a survey of all sections is first required to enumerate the number of residential and non-residential structures, the apparent simplicity of the single-stage cluster sampling design is reduced. In our previous paper, [3] we discuss this issue is more detail.

## Summary and Conclusions

We have developed and modeled two different but complementary approaches for stratified sampling in resource-limited environments. Their relative efficiencies have been discussed, and illustrated graphically and numerically. It does not seem likely that significant additional improvements can be achieved with respect to the stratification of the variable “persons per residential structure” demonstrated herein. Conversely, the single-stage cluster sampling method could well be the subject of additional research and application.

The stratification approach used for the latter was based on the partitioning of sections (clusters) into strata as a function of the number of residential structures per section. Alternative stratification variables could also be explored. As a hypothetical example, the section data available in this study encompasses 20 randomly-selected sections of the 68 sections comprising Bo City. Given data for all 68 sections, it would be possible to divide Bo City into a complete 68 section grid. Sections could then be assigned to strata as a function of the radial distance from the center of the city, or some other rule relating to geographical location or proximity.

### Answers to Key Questions

The objective of the current study was to examine methods for either re-estimating the population following a complete survey, or for estimating the population in a new environment under conditions which—for reasons of schedule or funding—preclude undertaking similar surveys. The ground truth data used for the simulations came from a larger field survey that collected data for the 20 municipal sections described in this paper [28–30]. The first method used proportional and Neyman-allocated optimal stratification, and the latter achieved a reduction in uncertainty of the population estimation of about 80% in 1,000 simulated sampling trials. For proportional allocation only, the simulations were also validated by comparing the estimates obtained using a stratified finite population bootstrap with comparable estimates using an unbiased Thompson-Horovitz estimator. The second method explored the use of single-stage cluster sampling. The uncertainty of the population estimates for the latter protocol was significantly improved by first stratifying the 20 sections into 4 strata as a function of section size (i.e., number of residential structures per section). If the total number of persons per section was used as the stratification variable, a further reduction in uncertainty was observed, but this variable may not be known prior to conducting a survey.

We can now briefly answer the 4 questions raised in the section “Proposed Analysis.”

1. If the 1,979 residential survey records are first partitioned into mutually-exclusive strata using “persons per residential structure” as the stratification variable, there is a reduction in uncertainty of about 80% relative to the estimate obtained using random sampling. The strata are cleanly separated by non-overlapping ranges of “persons per structure,” as shown in Fig 5. Because the variable “persons per residential structure’ must be known in advance, presumably from prior survey data, this protocol is potentially useful for re-estimating a population.
2. If the strata are created by partitioning the 20 *sections* into mutually-exclusive groups, using either residential structures or individual persons per section as the stratification variable, no statistically significant reduction in uncertainty is observed. The distributions of “persons per residential structure” overlap significantly between strata, and the strata are no longer well separated. Compare Fig 4 with Fig 5.
3. For 4-section single-stage cluster sampling, if the 20 sections are partitioned into mutually-exclusive strata by “total residential structures per section,” the uncertainty (H-T variance) of the population estimate is about 50% of the uncertainty for unstratified sampling. See Table 6 and Fig 6A.
4. If the sections are instead stratified by “total persons per section” the uncertainty of the population estimate is reduced to about 6% of the uncertainty of the unstratified case for single-stage cluster sampling. See Table 6 and Fig 6B.

### Future applications and research

For the single-stage cluster sampling, the sections were stratified by either total number of buildings per section, or by total persons per section. As an alternative, Bo could divided up into equal squares using a grid. There is a reasonably well-defined center of Bo, just are there are reasonably well-defined high-population-density centers that could be visually identified from aerial photographs of most cities. It is clear that if a grid was overlaid on a map of Bo, the cells farther from dense population areas would have fewer residential structures and a lower population density. If a Neyman stratification algorithm were to be applied, we would hypothesize that cells would be assigned to strata as a rough function of their distance from the center of the city. It would be interesting to compare the efficiency of this protocol for stratification with our existing results for single-stage cluster sampling, looking for possible improvement. At this time, we do not have sufficient data to test this hypothesis.

In summary, the ability to quickly estimate the total population size with reasonable precision in resource-limited environments can be of high value for demography, epidemiology, and health and social services research. The two approaches analyzed here are both of potential value in achieving these goals. Although the optimal stratification by residential occupancy is highly efficient, a single-stage cluster sampling protocol requires minimal data in advance, while minimizing the number of sections that must be surveyed.

## Supporting Information

S1 File Related manuscript [3]. (PDF)
