# Supplementary material for: Stratified Sampling of Neighborhood Sections for Population Estimation: A Case Study of Bo City, Sierra Leone
Source: PLoS One. 2015 Jul 15;10(7):e0132850. doi: 10.1371/journal.pone.0132850 (PMC4503693; doi:10.1371/journal.pone.0132850)
Supplement: S1 File — (PDF) [file pone.0132850.s001.pdf]

OPEN ACCESS Freely available online

PLOS | ONE

Methods for Determining the Uncertainty of Population Estimates Derived from Satellite Imagery and Limited Survey Data: A Case Study of Bo City, Sierra Leone

Roger Hillson[1¤], Joel D. Alejandre[1], Kathryn H. Jacobsen[2], Rashid Ansumana[3,4], Alfred S. Bockarie[3,4], Umaru Bangura[4], Joseph M. Lamin[4], Anthony P. Malanoski[5], David A. Stenger[5]*

1 Information Technology Division, Naval Research Laboratory, Washington, District of Columbia, United States of America, **2** Department of Global and Community Health, George Mason University, Fairfax, Virginia, United States of America, **3** Njala University, Bo, Sierra Leone, **4** Mercy Hospital Research Laboratory, Bo, Sierra Leone, **5** Center for Bio/Molecular Science and Engineering, Naval Research Laboratory, Washington, District of Columbia, United States of America

Abstract

This study demonstrates the use of bootstrap methods to estimate the total population of urban and periurban areas using satellite imagery and limited survey data. We conducted complete household surveys in 20 neighborhoods in the city of Bo, Sierra Leone, which collectively were home to 25,954 persons living in 1,979 residential structures. For five of those twenty sections, we quantized the rooftop areas of structures extracted from satellite images. We used bootstrap statistical methods to estimate the total population of the pooled sections, including the associated uncertainty intervals, as a function of sample size. Evaluations based either on rooftop area per person or on the mean number of occupants per residence both converged on the true population size. We demonstrate with this simulation that demographic surveys of a relatively small proportion of residences can provide a foundation for accurately estimating the total population in conjunction with aerial photographs.

Citation: Hillson R, Alejandre JD, Jacobsen KH, Ansumana R, Bockarie AS, et al. (2014) Methods for Determining the Uncertainty of Population Estimates Derived from Satellite Imagery and Limited Survey Data: A Case Study of Bo City, Sierra Leone. PLoS ONE 9(11): e112241. doi:10.1371/journal.pone.0112241

Editor: Abdisalan Mohamed Noor, Kenya Medical Research Institute - Wellcome Trust Research Programme, Kenya

Received January 31, 2014; **Accepted** October 7, 2014; **Published** November 14, 2014

This is an open-access article, free of all copyright, and may be freely reproduced, distributed, transmitted, modified, built upon, or otherwise used by anyone for any lawful purpose. The work is made available under the Creative Commons CC0 public domain dedication.

Funding: This work was funded by the Defense Threat Reduction Agency, Joint Science and Technology Office via contract to the United States Naval Research Laboratory. There is no past, present or future Intellectual Property associated with the work described in the paper, and none of the authors have any financial interests or conflicts in the outcome of the study. The funders had no role in study design, data collection and analysis, decision to publish, or preparation of the manuscript.

Competing Interests: The authors have declared that no competing interests exist.

* Email: david.stenger@nrl.navy.mil

¤ Current address: Independent consultant, Fairfax, Virginia, United States of America

Introduction

As Viel and Tran [1] have summarized, "Epidemiology is sometimes considered the science of denominators, because an accurate knowledge of the population at risk is a fundamental requirement for determining rates and deriving meaningful indicators of health status, health services, and health systems." Calculation of the rates of incidence of new disease or the prevalence of existing disease in a population or subpopulation depends on an accurate knowledge of the underlying population denominators, often stratified by age, sex, and even by levels of immunity or exposure [2,3]. Without adequate population denominator data, incidence and prevalence measures must be reported as counts rather than rates, which introduces a bias toward higher apparent risk in areas with higher population density [4]. Although maximum likelihood methods can now be used to estimate both the serial interval and reproductive number of an epidemic from counts of incident cases only [5,6], resource-limited countries often lack data for both the underlying population denominators and the baseline incidence rates. This is the situation addressed in this manuscript.

Our overarching goal with our simulations is to demonstrate approaches for combining readily available satellite imagery with small sample size field surveys as a means of efficiently estimating both population size (denominator data) and health states (numerator data). In this paper, we take a first step towards improving (denominator) measures by estimating total population in a West African urban area using remotely-acquired residential rooftop areas and demographic surveys. Specifically we ask "what number of residential surveys are needed to support a particular level of confidence in the estimated total population size, given a total number of residential structures or a total rooftop area?" We approach this question using a combination of total rooftop areas extracted from satellite imagery; a complete count of residents from 20 neighborhoods in Bo, Sierra Leone, West Africa, which we collected during a household health census; and simulated datasets sampled from our complete demographic dataset to represent small surveys of a subsample of residents.

Although our primary interests are in epidemiological and health care modeling, the simulation methods presented in this paper are not restricted to this domain since "denominator" data are essential for a variety of sectors. In resource-challenged environments, complete information about population size is often unavailable due to infrequent government census initiatives, rapid population growth in cities due to unplanned urbanization and rural-to-urban migration, political and other instability that prevents ongoing data collection and management, and the difficulties associated with securing the time and funding to conduct large-scale data collection activities. Rooftop areas,

readily acquired from satellite imagery, and household surveys for a limited number of residences may be combined with the algorithms developed in this paper to produce robust estimates of spatial population distributions.

Background

There are multiple applications for population data, including demographic profiling, the determination of disease incidence and prevalence, the mapping of access to health services, and the estimation of the number of refugees or displaced persons requiring emergency services. When combined with satellite imagery, survey data can facilitate the estimation of population size and population density.

Geospatial Information Systems (GIS) methods are increasingly being used for population estimation purposes. Wu et al. [7] distinguish between aerial interpolation methods that project census data onto a standardized population surface and statistical modeling methods that use socioeconomic variables (such as map layers for urbanicity, land use, built structures, and other physical or human geographic data) to calculate population estimates. Several research groups have used GIS to estimate population sizes. For example, the LandScan project, maintained by the Oak Ridge National Laboratory (ORNL), uses a dasymetric mapping technique that redistributes census-based population counts based on constraints imposed by geographic boundaries, the presence of physical structures, and/or public spaces, bodies of water, and other spatial constraints [8]. The Gridded Population of the World (GPW) and the derivative Global Rural-Urban Mapping Project (GRUMP), both distributed through NASAâ€s Socioeconomic Data and Applications Center (SEDAC), which is hosted by the Center for International Earth Science Information Network (CIESIN) at Columbia University have developed novel methods for estimating the population of urban areas, such as analyzing nighttime satellite images to map city footprints [9] (see [10,11] for applications).

In this paper, we used spatially-directed surveying to gather field data about households in neighborhoods of interest. Using ground truth information about building use and residential occupancy in conjunction with imagery-derived estimates of rooftop areas, we applied a population bootstrap algorithm to estimate the uncertainty of the estimated population as a function of survey sample size. The two estimators used are based on average occupancy per residential structure, or average rooftop area per person. Prior studies have reported findings using comparable methods. Checchi et al. [12] used an occupancy-based measure to estimate the number of displaced persons at 11 different sites. The population was estimated as the product of (1) the *number of structures* and (2) the average *occupancy per structure*, as inferred from published reports. The local population may be overestimated in situations where buildings and tents were not physically distinct from one another, or occupied structures are concealed by trees. Aminipouri [13] used rooftop extraction methods to estimate the total rooftop area for three different slum areas in Tanzania. The total population was estimated as the product of (1) the *total rooftop area* and (2) the *number of persons per rooftop area*, based on an assumption that the majority of dwellings were residential. We compare both estimation results with field data from a household census, and also attempt to quantify the relationship between the uncertainty of the population estimate and the survey size.

Methods

Survey Methodology and Dataset Development

Terminology. In Bo, Sierra Leone's second largest city, a "section" is a formal municipal area with defined boundaries. Inside a section a variety of building structure types may be observed in satellite imagery. We divided structure types into two major categories, residential and non-residential. Residential structures include all buildings where persons sleep at night. Small ancillary structures such as carports, outdoor kitchens, storage sheds, chicken coops, latrines, and small sheds used for micro-enterprise activities which are located on residential properties but not used for sleeping were excluded when we delineated residential structures and rooftop areas. A household was defined as a person or group of persons identifying themselves as a family unit and residing within one structure. Each residence comprises the sleeping space for one or more households. The non-residential category includes governmental, commercial, nonprofit organizational, and religious structures.

Survey Methods and Data Assembly. Our creation of a complete municipal map for Bo, Sierra Leone (central coordinates: 7.959°, −11.740°), using participatory GIS methods has been described elsewhere [14]. Long-term residents and city officials were consulted throughout the mapping and validation process to ensure the accuracy of section boundaries and other map features. Sections in Bo are divided both by natural topographies, such as marshy areas, and by man-made structures such as roads. The central area, which is older, has a more planned layout than the sections toward the edges of the city, where the growth was more informal. Figure 1A shows the satellite image of Bo with the boundaries of sections described in this study marked (red lines). Figure 1B shows the same sections in relation to the overall boundaries of Bo. For surveying, the first two sections (Kulanda Town and Njai Town) were selected for convenience due to proximity to the Mercy Hospital Research Laboratory (MHRL) building, which is located in Kulanda Town. There were 68 sections in Bo at the time of the survey. After surveying Kulanda Town and Njai Town, a random number generator was used to select 18 of the remaining 66 sections for inclusion in the expanded survey. In total, we surveyed 20 of the 68 sections. All field surveyors – MHRL staff and graduate students from Njala University – were residents of the city of Bo. Prior to beginning data collection, all surveyors and interviewers completed several days of training, including instruction on geographic data collection (determining Global Positioning System (GPS) coordinates with a handheld device, a Garmin GPSMAP 62 series), interviewing techniques, and research ethics and regulations. The two sections adjacent to MHRL were surveyed in April 2010. The remaining sections were surveyed between November 2010 and February 2011.

Contact with the households occurred in two stages. First, one adult representative male or female from each residential structure was interviewed about (1) the number of households in the residence, (2) the number of persons in the informant's household, and (3) the age and sex of each person in his or her household. Many residential structures were home to multiple households, even if the residence was a modestly sized structure. Households were usually related, but maintained some independence in function. For example, if two adult siblings were both living in the residence with their minor children, then they were generally reported as being two separate households. When two or more households lived within the same residence, a representative of each household was asked to provide information about the composition of his or her household. Second, the enumerator

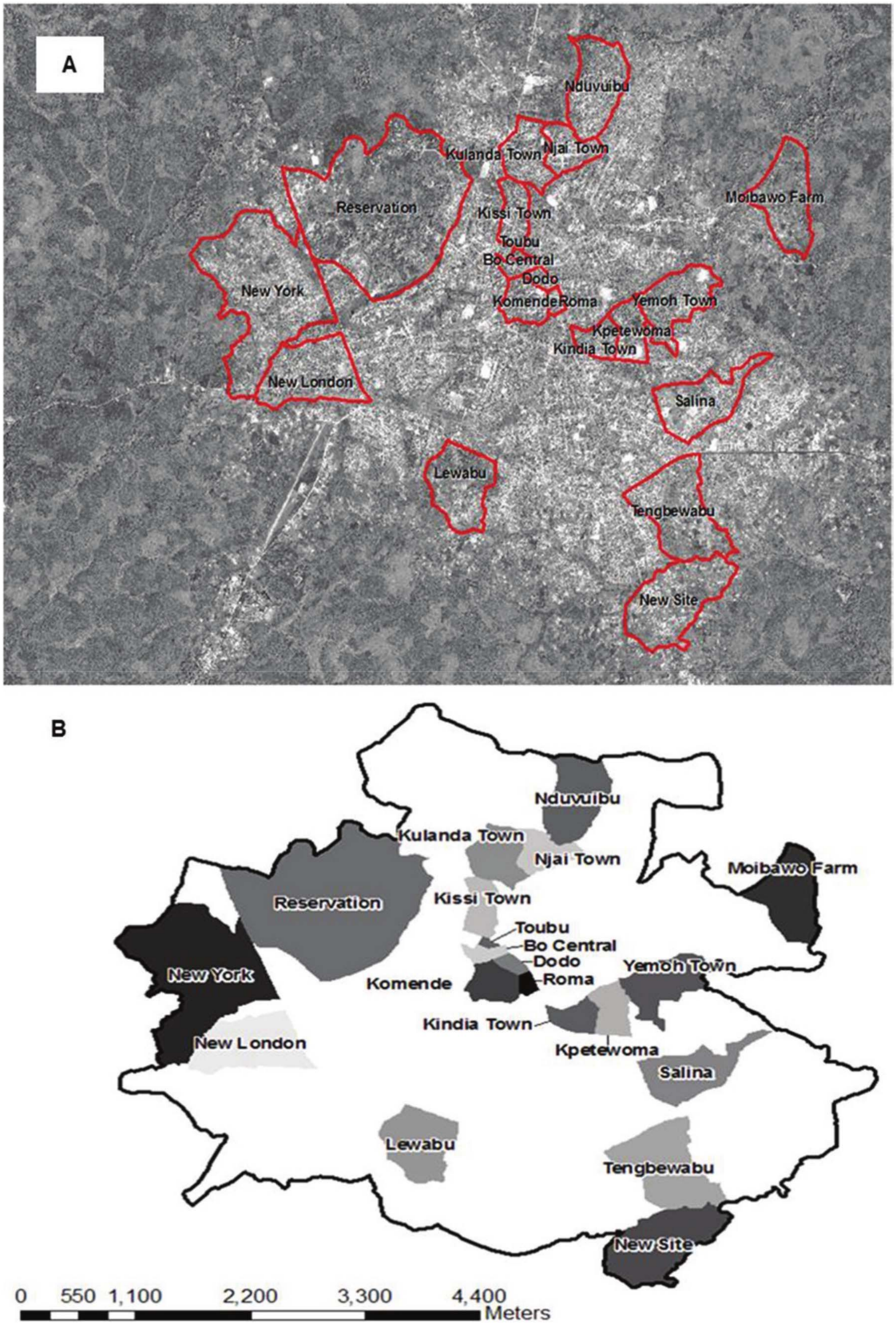

Figure 1. Aerial map of Bo city and municipal sections. (A) Aerial map of Bo, Sierra Leone showing municipal sections (red lines) described in this study and (B) Graphical representation of the same. (Image copyright 2010 DigitalGlobe NextView License. Used with permission.)
doi:10.1371/journal.pone.0112241.g001

Table 1. Summary of survey data for 20 sections of Bo.

Section	Area (km^2)	Structures	Residential Structures*	Households*	Persons*	Population Density†
Bo Central	0.07	103	33	51	273	high
Dodo	0.05	88	26	85	597	high
Kindia Town	0.15	278	102	206	1,160	high
Kissi Town	0.20	287	154	400	2,490	high
Komende	0.20	258	56	175	1,103	high
Kpetewoma	0.20	105	46	94	640	medium
Kulanda Town	0.29	314	197	637	3,882	high
Lewabu	0.48	117	105	170	879	low
Moibawo Farm	0.50	43	17	22	135	low
Nduvuibu	0.49	343	205	439	2,552	medium
New London	0.60	495	208	498	2,873	medium
New Site South (New England)	0.69	194	136	190	1,248	medium
New York	1.51	605	116	176	1,088	low
Njai Town	0.22	269	127	388	2,298	medium
Reservation	2.33	252	66	86	637	low
Roma	0.04	52	4	22	139	high
Salina	0.47	231	59	110	580	low
Tengbewabu	0.68	233	136	185	1,068	low
Toubu	0.02	46	34	88	454	medium
Yemoh Town	0.40	284	152	289	1,858	medium
Total	—	4,597	1,979	4,311	25,954	—

Complete residence and household survey data for 20 municipal sections of Bo, showing the area, the total number of structures and the number of residential structures, households and persons per municipal section.
*From survey data collected from all occupied structures in the section.
†high density: $\geq 11{,}000$ residents per km^2; medium density: 5,500 to 10,999 residents per km^2; low density: $<5{,}500$ residents per km^2.
doi:10.1371/journal.pone.0112241.t001

sought to interview all mothers of any age who were living in the residence. Only the first of the two stages are relevant for the current analysis.

Handheld GPS units were used to obtain geographical coordinates of all residential structures in the 20 municipal sections. During this process, field staff distinguished between residential and non-residential structures [14]. For two sections where the types and dimensions of all structures (residential and non-residential) were quantized, field surveyors marked the coordinates of all non-ancillary structures and noted whether they were residential or non-residential. Satellite imagery with 0.5-meter resolution (Digital Globe, Satellite: WorldView-1, Image date: 7 February 2010) was used in GIS software, ArcGIS (ESRI, Inc. Redlands, CA) or Quantum GIS (QGIS; www.qgis.org), to digitize rooftops so that individual areas could be determined. The area for each rooftop was calculated only from the two-dimensional coordinates measurable from satellite imagery, irrespective of the total area that would be calculated using full three-dimensional coordinates.

Protection of Human Subjects. All data collection involving human subjects was approved by the institutional review boards of Njala University, George Mason University, and the U.S. Naval Research Laboratory. Written informed consent was obtained from each household representative who participated in the survey, all of whom were adults.

Table 2. The subset of Bo residences with known occupancies and rooftop areas.

Section	Residential Structures with Known Rooftop Areas	Total Residential Structures in Section	Persons in Residential Structures with Known Rooftop Areas
Bo Central	25	33	241
Komende	53	56	1,061
Kulanda Town	193	197	3,809
Njai Town	127	127	2,298
Reservation	66	66	637
Total	464	479	8,046

Subset of structures by neighborhood for which data were available for both the rooftop area and total number of residents in five municipal sections.
doi:10.1371/journal.pone.0112241.t002

Table 3. Summary of the four datasets used for the simulation.

Dataset	$DS01$	$DS02$	$DS03$	$DS04$
Brief description	All residential structures in the 20 sections	DS01 subset: 5 sections with rooftop areas	Njai Town Composite (Mashup)	Reservation Composite (Mashup)
Total number of residential records (Tr)	1,979	464	127	66
Total number of non-residential records (Tn)	Not found	Not found	142	186
Total number of structures in dataset ($Ts = Tr + Tn$)	1,979	464	269	252
Total rooftop area (m^2) of residential structures (Ar)	Not found	83,605	21,924	16,857
Total rooftop area (m^2) of non-residential structures (An)	Not found	Not found	15,434	30,024
Total rooftop area (m^2) of residential and non-residential structures (As)	Not found	Not found	37,358	46,881
Number of sections in dataset	20	5	1	1
Total number of individuals	25,954	8,046	2,298	637
Total number of households	4,311	1,313	388	86
Average number of individuals per residential structure	13.1 ($\sigma = 9.1$)	17.3 ($\sigma = 11.7$)	18.1 ($\sigma = 10.4$)	9.7 ($\sigma = 4.7$)
Average rooftop area (m^2) per residential structure	Not found	180.2 ($\sigma = 88.7$)	172.6 ($\sigma = 66.9$)	255.4 ($\sigma = 4.7$)
Average rooftop area (m^2) per non-residential structure	Not found	Not found	108.7 ($\sigma = 70.6$)	161.4 ($\sigma = 91.7$)
Average residential rooftop area (m^2) per individual resident	Not found	16.7 ($\sigma = 18.2$)	13.7 ($\sigma = 13.0$)	32.2 ($\sigma = 22.8$)
Number of records with digitized rooftop areas	464 (5/20) sections	464 (of 479) records with rooftop areas	269	252

Summary of the four datasets used for the simulation.
doi:10.1371/journal.pone.0112241.t003

Bo City Dataset Construction. Complete survey data were collected for 20 of the 68 sections (neighborhoods) in Bo (Table 1). This ground truth Dataset 01 (*DS01*) include survey records for 1,979 residential structures, representing a total population of 25,954 individuals. Five sections – Bo Central, Komende, Kulanda Town, Njai Town, and Reservation, which collectively are home to 479 residential structures and 8,046 individuals (*DS02*) – were selected for manual digitization of the rooftop areas for all of their residential structures (Table 2). For two sections, Njai Town (*DS03*) and Reservation (*DS04*), the rooftop areas of the non-residential structures were also digitized, and composite mashup datasets were constructed that contain records for both residential and non-residential structures (Table 3). Reservation, originally established as a housing area for government officials, was selected as an example of a section with large lots and a relatively high proportion of non-residential buildings. Njai Town was selected as an example of a densely populated residential neighborhood with few commercial structures. The differences between these two sections allowed us to better compare and evaluate population estimation approaches. Cross-matching of the residential rooftop areas to surveyed households was used to identify residential structures; the remaining structures were classified as non-residential. By definition, a non-residential record in *DS03* and *DS04* has a digitized rooftop area and the number of individuals and households is set to 0. Figure 2 summarizes the 4 datasets and all the simulations that were run.

Population Estimation Methods

Two Population Estimators. Using the GIS data summarized above and spatially-linked demographic data we collected in Bo, we are able to simulate the estimation of the population (and the associated uncertainty) as a function of survey size using two different population estimators:

1. An occupancy-based estimator, which is proportional to the product of (1) the average number of persons per residential structure and (2) the estimated total number of residential structures (Tr).
2. A rooftop area-based estimator, which is proportional to the product of (1) the average number of persons per m^2 of rooftop area and (2) the estimated total rooftop area of all of the residential structures (Ar).

For n surveyed residences of index i and number of individual persons IND_i in the ith residence, and the estimated total population $\hat{P}$ based on the total number of residences is given by:

$$Estimated\_Total\_Population = \hat{P} = \left(\sum_{i=1}^{n} \frac{IND_i}{n}\right) * Total\_Number \quad (1)$$

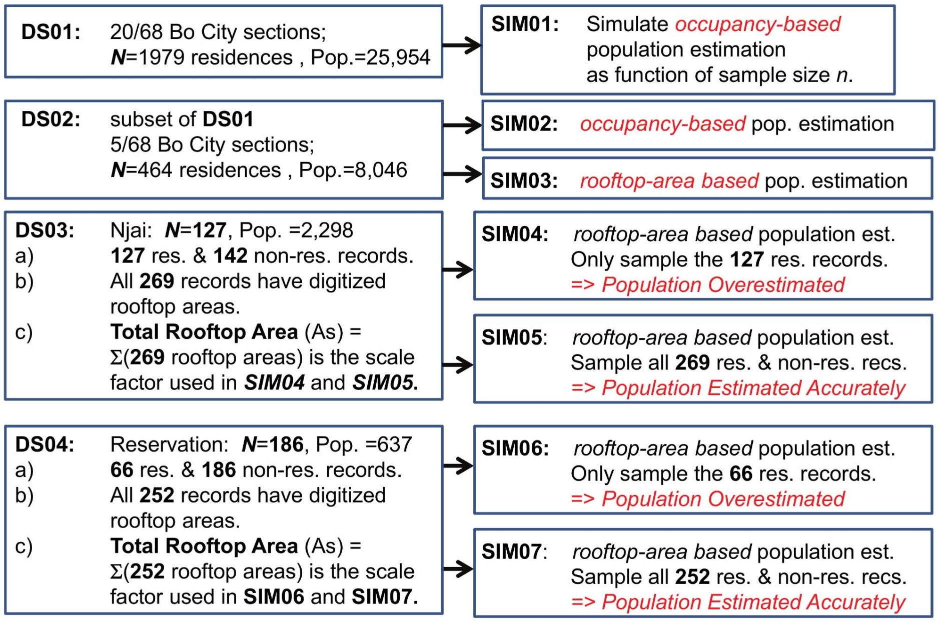

Figure 2. Flowchart illustrating datasets and simulations.
doi:10.1371/journal.pone.0112241.g002

In Equation (1), $Total\_Number$ is the scale factor for the population estimator, and is either the $Total\_Number$ of residential structures, or the $Total\_Number$ of residential and non-residential structures. This scale factor is required because the total number of residence structures $Tr < Ts$, where Ts is the total number of residential plus non-residential structures. If only residential structures are included in the survey, then $Total\_Number = Tr$. If the survey includes both residential and non-residential structures, then $Total\_Number$ should be set equal to Ts, rather than Tr.

Alternatively, the population may be estimated on the total area of the residence rooftops:

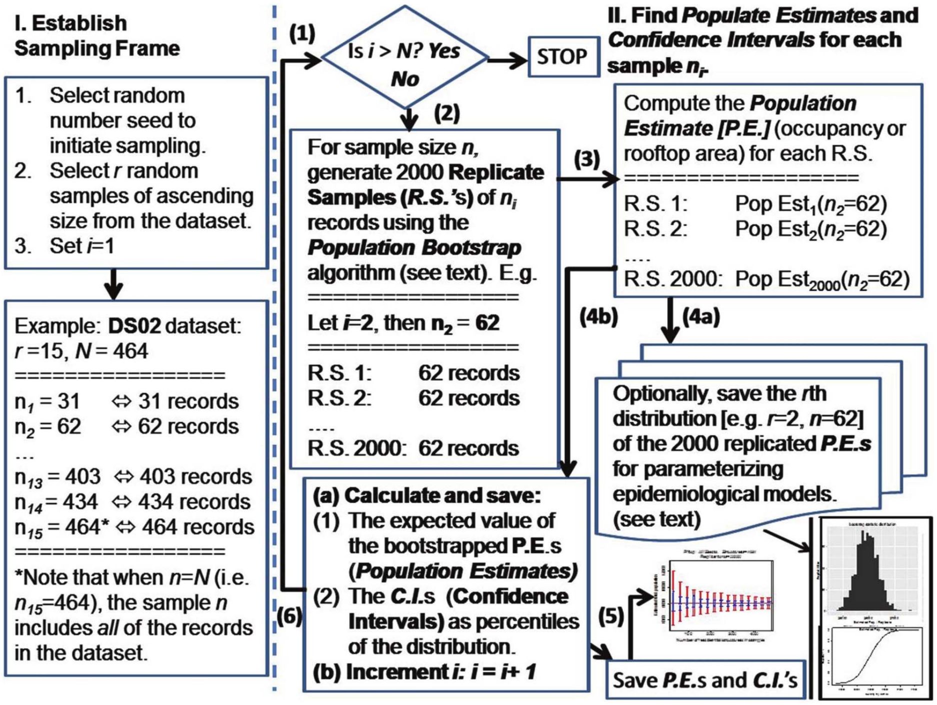

Figure 3. Flowchart for population bootstrap.
doi:10.1371/journal.pone.0112241.g003

Table 4. Summary of the 6 simulations and parameters.

Simulation	$SIM01$	$SIM02$	$SIM03$	$SIM04$	$SIM05$	$SIM06$	$SIM07$
Number of records	1,979	464	464	269	269	252	252
Record type(s)	Residential records only	Residential records only (with rooftop areas)	Residential records only (with rooftop areas)	127 residential records	127 residential & 142 non-residential records	66 residential records	66 residential & 186 non-residential records
Number of Sections	20	5	5	1 (Njai Town)	1 (Njai Town)	1 (Reservation)	1 (Reservation)
Converges to correct population estimate?	Yes	Yes	Yes	No	Yes	No	Yes
Estimator used	Occupancy	Occupancy	Rooftop	Both	Both	Both	Both
Estimator scale factor used	$Tr=1{,}979$ residential structures	$Tr=464$ residential structures	$Ar=83{,}605\ m^2$	$Ar=21{,}924\ m^2$	$As=37{,}358\ m^2$	$Ar=16{,}857\ m^2$	$As=46{,}881\ m^2$
Number, increment Δ, and *sequence* of the simulated survey samples	15 steps, $\Delta=132$: [132, 264, ..., 1716, 1848, 1,979]	15 steps, $\Delta=31$: [31, 62 ... 403, 434, 464]	15 steps, $\Delta=31$: [31, 62 ... 403, 434, 464]	16 steps, $\Delta=8$: [8, 16, ..., 112, 120, 127]	15 steps, $\Delta=18$: [18, 36, ..., 234, 252, 269]	16 steps, $\Delta=4$: [4, 8, ..., 56, 60, 66]	15 steps, $\Delta=17$: [17, 34, ..., 221, 238, 252]

Summary of the six simulations run, including all of the scale factors used.

doi:10.1371/journal.pone.0112241.t004

$$Estimated\_Total\_Population = \hat{P} = \left(\frac{\left(\sum_{i=1}^{n} IND_i\right)}{\left(\sum_{i=1}^{n} Res\_Area_i\right)}\right) * Total\_Area \tag{2}$$

In Equation (2), $Total\_Area$ is the scale factor for the population estimator. $Total\_Area$ is either the total rooftop area of the residential structures, or the $Total\_Area$ of the residential and non-residential structures. This scale factor is required because the total rooftop area of residence structures $Ar < As$, where As is the total rooftop area of (residential plus non-residential) structures. If only residential rooftop areas are included in the survey, then $Total\_Area = Ar$. If the survey includes both residential and non-residential structures, then $Total\_Area$ should be set to As rather than Ar.

A population bootstrap algorithm [15] (pages 93–94) is used to estimate the uncertainty of the two estimators as a function of survey size. We will demonstrate that the population of the pooled survey sections can be accurately estimated from small samples of survey participants, given reasonable values for specific parameters such as the total number of residential buildings or the total rooftop area of all residential buildings. The latter parameters can be partially derived from aerial imagery, but residential and non-residential structures cannot be readily distinguished using the imagery alone.

Figure 3 is a flowchart of the bootstrap simulation approach used. Two estimators are tested, the occupancy-based population estimator and the rooftop area-based population estimator. For a given dataset, and for each sample size n, 2,000 replicate bootstrap samples are created (step 2), using the population bootstrap algorithm. The desired population estimate is calculated for each replicate (step 3), along with the associated confidence intervals. The individual 2,000 bootstrap replicate samples themselves can be saved, if desired (step 4a). Alternative estimates of the population and the associated uncertainty can be made from the distributions of replicated samples, as required to parameterize external epidemiological models as a function of sample size. The expected bootstrap estimate of the population, and the associated confidence levels (0.50 and 0.95), are always calculated (step 4b) and saved (step 5). If surveys for all of the sample sizes have not been completed, this process will proceed through the next iteration (step 6).

The estimated total population is the mean value of the 2,000 individual population estimates [15]. The confidence levels (0.50 and 0.95) for the estimate of the total population were calculated as the equal-tailed two-sided bootstrap percentile intervals [16]. The basic bootstrap methodology is summarized in [17], and percentile methods are discussed in [15,18]. The specific samples selected from the pool of 1,979 residences were dictated by the choice of the initial random number seed used for the sampling function, as well as the sequence of contingent bootstrap replicates. Each of the 2,000 replications for a sample of size n requires the random selection of 2,000 samples, each of size n. A random number seed is a number, or vector of numbers, that is used to initialize the pseudorandom number generator used to randomly select the survey records in step 3 of Figure 2. This initial seed was always explicitly specified and saved, which facilitated the uniform replication of any of the simulations presented in this analysis. Changing the random number seed will result in a different sequence of bootstrap replicate samples, and the output from the bootstrap algorithm will vary, as will be discussed.

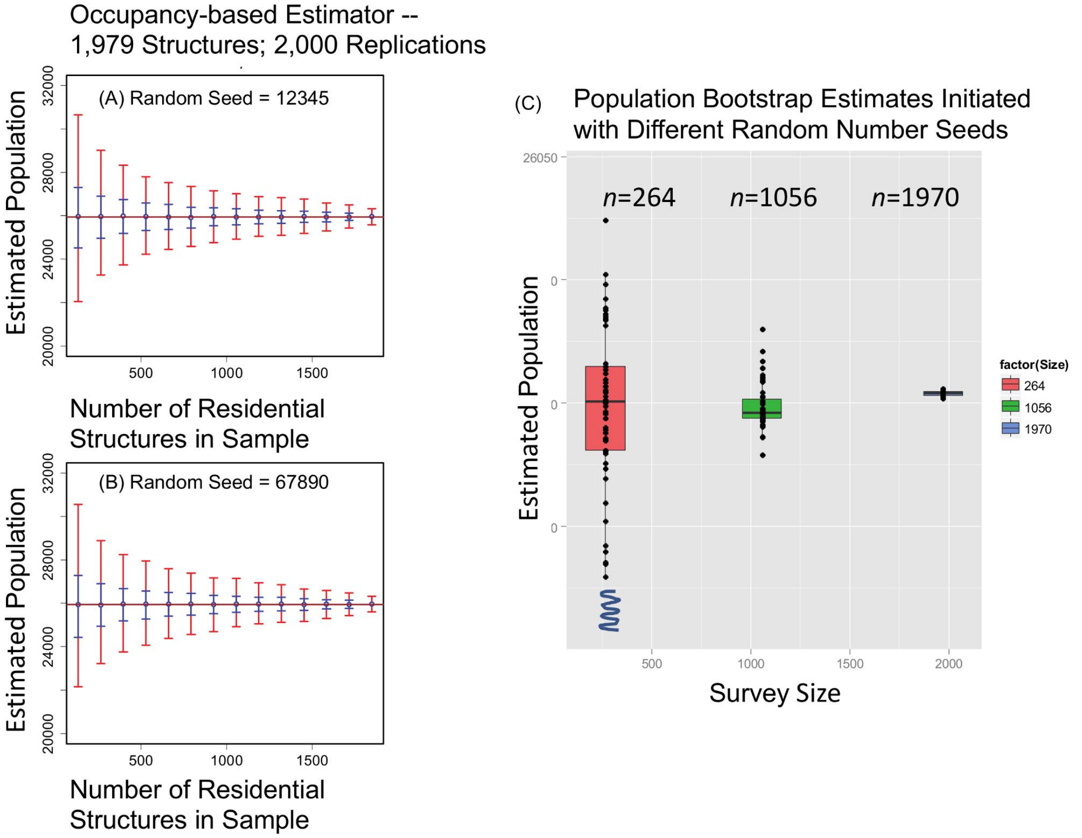

Figure 4. Occupancy-based bootstrap population estimations parameterized by sample size and random number seed. Results for (A) random number seed =12345 and (B) random number seed =67890. In both panels, the brown line is the total population count $P = 25{,}954$, the number of individuals counted in the 1,979 residences surveyed for *DS01*. The 0.50 CIs is shown in blue and the 0.95 CI in red. (C) A quartile boxplot shows the variation in the population bootstrap estimates $\hat{P}$ for 25 replicate samples initiated with different random number seeds.
doi:10.1371/journal.pone.0112241.g004

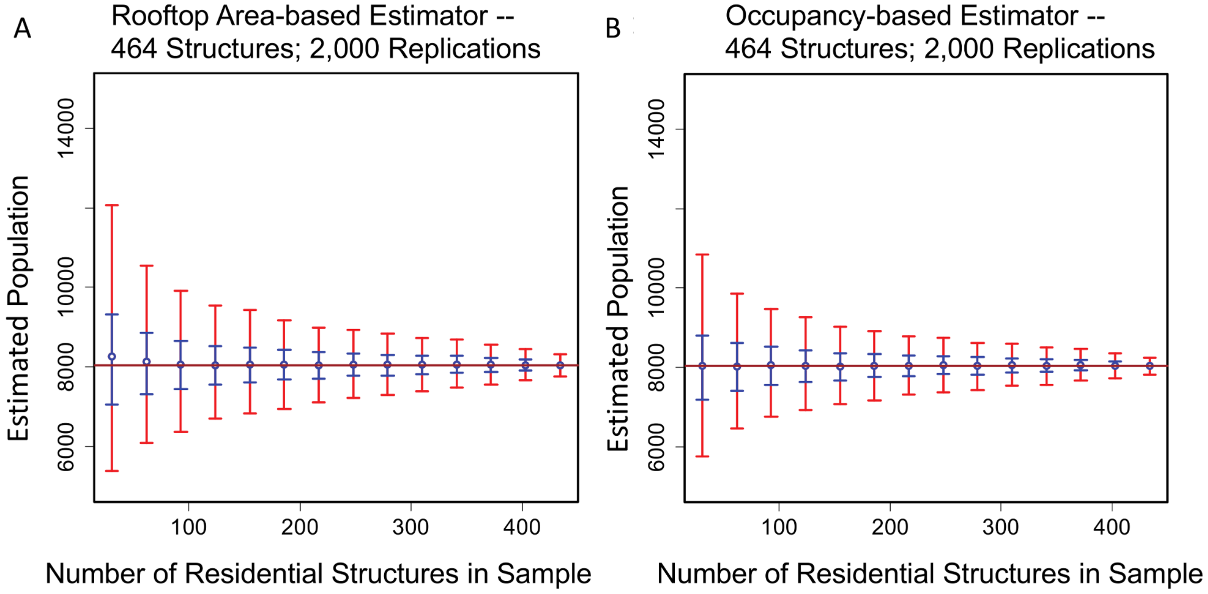

Figure 5. A comparison of occupancy-based and rooftop area-based bootstrap population estimators. (A) Occupancy-based population estimations and (B) rooftop area-based population estimations with 0.50 CIs (blue) and 0.95 CIs (red). There are 464 residences (*DS02*) and 2,000 bootstrap replications per sample. The confidence intervals (CIs) were calculated using bootstrap percentiles.
doi:10.1371/journal.pone.0112241.g005

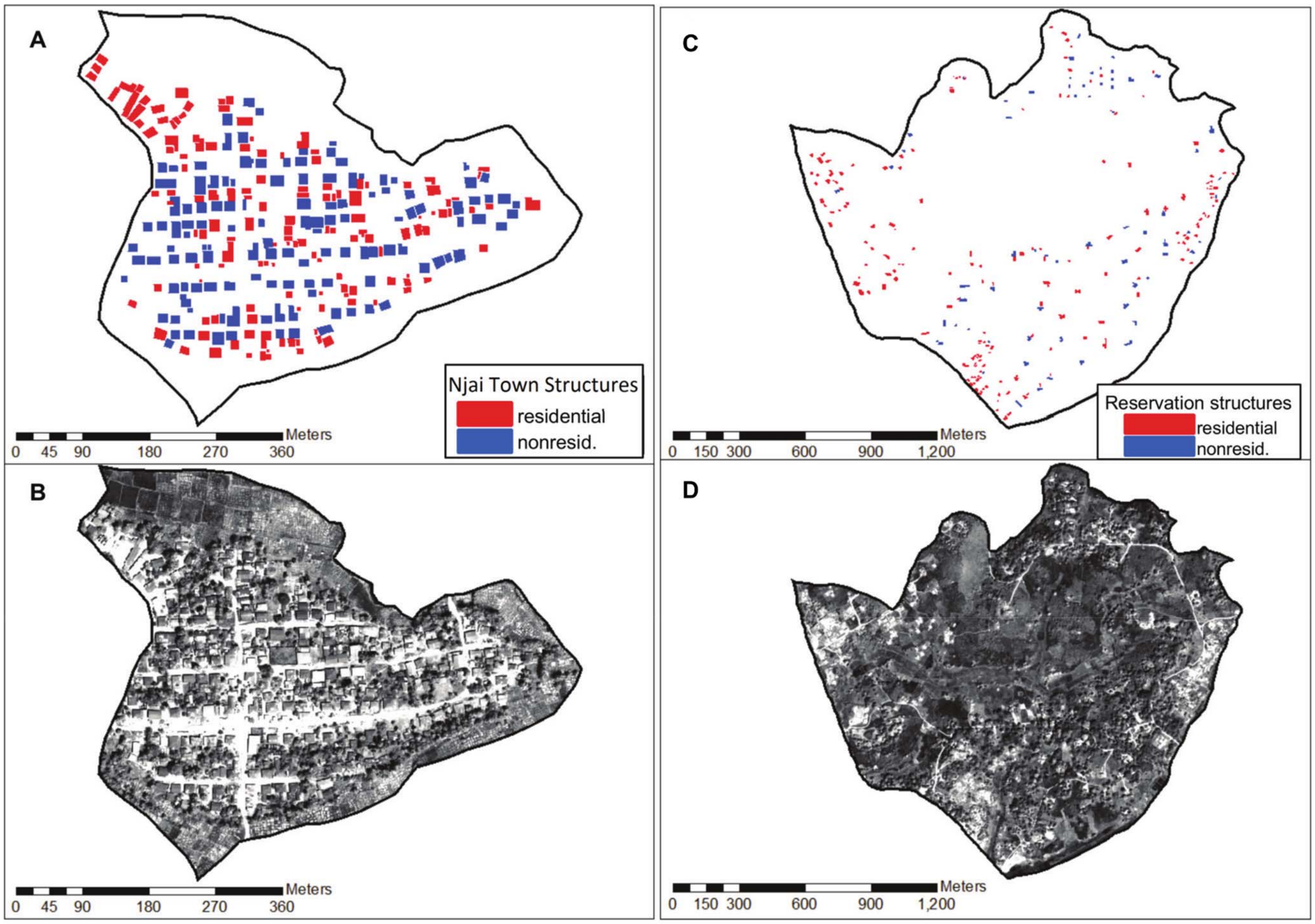

Figure 6. Residential and non-residential structures in Njai Town and Reservation. (A) Njai Town residence structures (blue) and non-residential structures (red), (B) Njai Town satellite imagery, (C) Reservation residential structures (blue) and non-residential structures (red), and (D) Reservation satellite imagery. When comparing either the maps or imagery for Njai Town and Reservation, note the differences in the map scales, which create the illusion that the structures in Reservation are smaller, although the average residential rooftop area in Reservation (255 m^2) is actually greater than that of Njai Town (172 m^2). The total land areas for Njai Town and Reservation are 0.21 km^2 and 2.33 km^2, respectively. (Image copyright 2010 DigitalGlobe NextView License. Used with permission.)
doi:10.1371/journal.pone.0112241.g006

Table 3 shows the values for Tr, Ts, Ar, and As as obtained for the 20- and 5-section aggregates and for Njai Town and Reservation individual sections. If any non-residential areas or structures were identified in satellite imagery, they were excluded from consideration. For the 20 and 5 section aggregates, only data for Tr were collected. For both Njai Town and Reservation sections, Tr, Ts, Ar, and As were completely delineated.

The total rooftop area As can be estimated from aerial imagery without distinguishing between residential and non-residential structures. If the ratio of Ar/As can be estimated, perhaps by collaboratively analyzing the aerial imagery and/or participatory GIS maps with local residents, Ar can then be calculated. A similar calculation yields Tr:

$$A_r = (A_r/A_s) \times (A_s) \quad (3)$$

$$T_r = (T_r/T_s) \times (T_s) \quad (4)$$

The Population Bootstrap Algorithm. If we had a single sample n, drawn from a larger unknown population, then a conventional bootstrap could be used for estimating the uncertainty. In this case, we would repeatedly resample without replacement from n, and determine the expected value of the estimator on the distribution of replicated samples – 2,000 replicates, in our examples. In our current study, we are drawing ascending samples of size n from a previously measured finite population of size N. More specifically, we are resampling from a finite population of either 464 or 1,979 houses (N) by drawing increasing samples of size n. The conventional bootstrap algorithm would fail to properly account for the decrease in the uncertainty (confidence intervals) that occurs as the sampling fraction (n/N) becomes large, and the number of possible samples n that can be drawn from N decrease as $C(N,n)$. The chapter on finite population sampling in Bootstrap Methods and Their Applications by Davison and Hinkley summarizes this issue succinctly: "The key difficulty with the ordinary bootstrap is that it involves with-replacement samples of size 'n' and so does not capture the effect of the sampling fraction, which is to shrink the variance of an estimator." [15].

Davison and Hinkley suggest four methods to correct for this effect: (1) modifying the sample size to match the estimator variance (2) matching the original sample size while maintaining

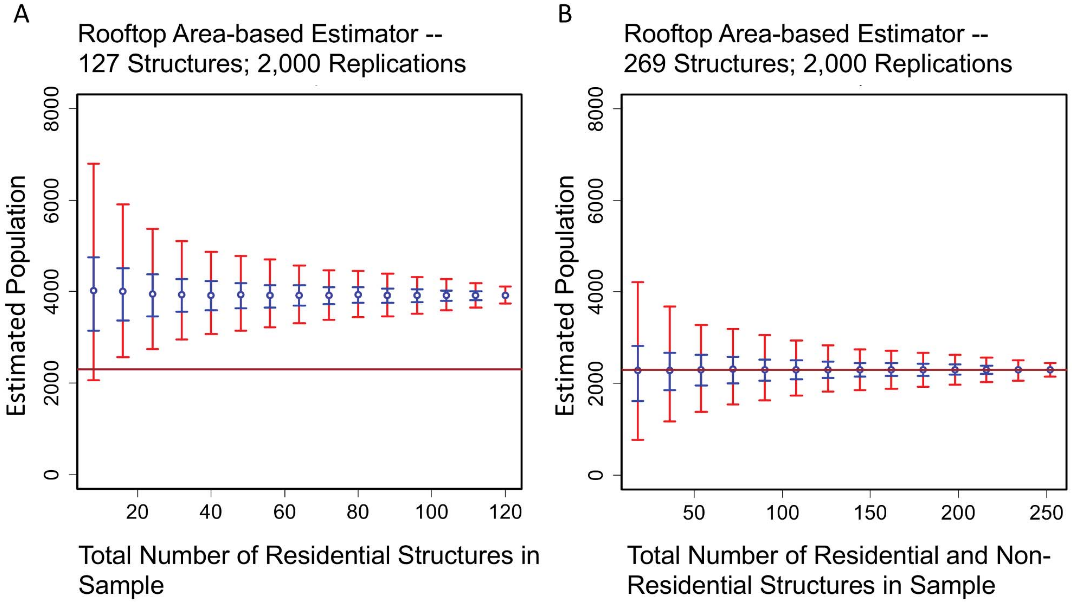

Figure 7. Rooftop area-based population estimations for Njai Town using invalid and valid scale factors. (A) Non-convergent rooftop area-based population estimate with 0.50 (blue) and 0.95 CIs (red). Samples were drawn from the Njai Town dataset of 127 residences only, but the scale factor $Total\_Area$ was set to the rooftop area for all 269 structures (As). (B) Convergent rooftop-based population estimate with 0.50 (red) and 0.95 (blue) CIs. Samples were drawn from the Njai Town dataset of 269 residential and non-residential structures, and $Total\_Area$ was set correctly set to As. Source: residential survey data and digitized rooftop areas, $As = 37{,}358\ m^2$.
doi:10.1371/journal.pone.0112241.g007

the sampling fraction - the mirror-match bootstrap, (3) the superpopulation bootstrap, and (4) the population bootstrap. In a resampling analysis of longitudinal United States city data, the authors conclude (page 97) "that the population and super-population bootstraps are the best of those considered here" [15]. The population bootstrap is easier to implement and executes faster than the superpopulation bootstrap, and is the algorithm used here.

Let $R = 2{,}000$. For each of the R bootstrap replicates, execute the following steps:

1. Randomly select without replacement a sample y_n $[y_1, y_2,\ldots, y_n]$ of n records from a dataset of size N. (For example, let $n = 264$ and $N = 1{,}979$.)
2. Create an empty vector Y^* of length N.
3. Copy $f = truncate(N/n)$ consecutive copies of y_n into Y^*, which now contains n*f records.

(a) for example, $f = truncate(1{,}979/264) = truncate(7.49) = 7$
(b) $n^*f = 7^*264 = 1{,}848$ records

4. Select $m = N\text{-}n^*f$ records without replacement from y_n, and append these records to Y^*, which will now contain N records.

(a) $m = 1{,}979–1{,}848 = 131$ records
(b) $n^*f{+}m = 1{,}848{+}131 = 1{,}979$ records $= N$

5. Select the population bootstrap replicate by randomly selecting n records without replacement from the N records in Y^*.
6. Repeat steps 1 to 5 to create R bootstrap replicates. In our examples, $R = 2{,}000$. Note that each of the 2,000 replicates has the same sampling fraction (n/N). The estimate is exact when $n = N$.

Results

Figure 2 and Table 4 summarize the simulations executed, and the datasets used.

1. *SIM01*. Figure 4AB shows the required convergence [19] of the occupancy-based population estimator as a function of the $N = 1{,}979$ residential structures in *DS01*. The variance (uncertainty) decreases uniformly as the sample size increases. The confidence intervals (CIs) are illustrated by the blue (0.50 CI) and red (0.95 CI) bars. The otherwise identical simulations were initiated with two different random seeds. Changing the random number seed will result in a different sequence of bootstrap replicate samples, and the estimated population $\hat{P}$ as a function of sample size n will vary. Using a different seed has little impact on the variance (CIs) but does result in modest variation in the estimated population $\hat{P}$ as a function of sample size, as shown in the quartile boxplots in Figure 4C. Note that the expanded Y-axis exaggerates the absolute variation relative to the measured value of the population.
2. *SIM02* and *SIM03*. The occupancy-based and rooftop-area based sequences of sample size simulations for dataset *DS02* ($N = 462$) are compared in Figure 5. Both estimators converge properly. The uncertainty of the occupancy-based estimator is about 20% less than for the rooftop-based estimator for both the 0.95 and 0.50 confidence intervals. For a given sample size n and confidence interval, the corresponding confidence interval is defined as the difference between the upper and lower confidence limits. For each estimator, and for each of the two confidence intervals, 14 confidence intervals were calculated. A two-tailed paired t test between the paired differences was statistically significant for both confidence levels ($p < 0.001$).

3. *SIM04* and *SIM05*. Figure 6 shows the imagery and map of the Njai Town and Reservation sections. Residential structures are denoted in red; non-residential structures are denoted in blue. Figure 7 compares the rooftop-area population estimator under two different scenarios, both using *DS03*. In SIM04 only the 127 residential records are sampled – that is, all records drawn have 1 or more individuals as residents – but the total rooftop area As is equal to the rooftop area of the combined residential and non-residential structures ($As = 37{,}358\ m^2$). This results in a total population estimate for Njai Town that is much greater than the true value. In *SIM05*, the population estimate draws on both residential and non-residential structures, so in some records the number of individuals is 0. This time the estimator converges correctly.

Thus, we have defined two different rooftop area-based protocols that can be used to estimate the population, with analogous protocols defined for the occupancy-based simulation:

1. Include both residential and nonresidential structures in the n records sampled for each survey size, using the scale factor As, knowing that many of the sampled records will have a population of 0.
2. Use the scale factor Ar, where $Ar < As$, and sample residential records only, as was done in *SIM02*.

Both methods will converge to the correct population estimate. For brevity, additional results are not graphed, but comparable findings were demonstrated using the occupancy-based population estimator with both the Njai Town and Reservation datasets *DS04* (simulations *SIM06* and *SIM07*).

Discussion

Occupancy-Based Estimation vs. Rooftop Area-Based Estimation

Based on the uncertainty functions, the occupancy-based population estimator is to be preferred. Both the occupancy-based and rooftop-area based estimators converge properly, with decreasing uncertainty as the sample size increases.

Determining which estimator also poses pragmatic considerations, apart from the relative technical merits of the estimators. Consider the following three scenarios:

1. Assume that high-resolution imagery is available, and automated procedures can be applied to extract rooftops and estimate their total area As. In this scenario the survey data can be represented spatially and coded to the location of the surveyed structures, if required. (We have not attempted to do so here, as mapping the survey data onto specific residential structures would violate our confidentiality agreement with participants.) If the survey data include both residential and non-nonresidential records, the population can be estimated as shown in Figure 5, using either the occupancy-based or rooftop-area based estimator. If the survey data are only for residential structures, either Ar or Tr must be estimated for the scale factor (Table 3), perhaps as a percentage of the measured values for As or Ts.
2. Assume that the individual residential structures can be resolved and counted automatically in the aerial imagery, but the rooftop areas cannot be successfully extracted. In this case, only the occupancy-based population estimator can be used, because the residential rooftop area Ar cannot be estimated.
3. Assume that individual structures cannot be resolved in the imagery, but it is possible to estimate the total rooftop area As in the region of interest. This may occur when using low-resolution imagery in a densely populated area. In this scenario, the rooftop-area based estimator alone is applicable, provided that either (1) the survey data include proportional numbers of residential and non-residential records, and As is the correct scale factor or (2) the survey data include residential records only, but Ar can be estimated as a percentage of the measured total rooftop area As.

Delineating Structures and Rooftop Areas. In practice, the total rooftop area and/or the total number of individual residences within a large area will likely be extracted from aerial imagery using automated methods, rather than manually, as was done here. Relevant methods for extracting buildings from aerial imagery are described in numerous publications (for example, see [13,20,21]). Object based image analysis (OBIA) provides a particularly powerful set of methods; see [22] for a review of OBIA and a discussion of available commercial tools such as eCognition [23]. Given our current data, it would be instructive to use a commercial program to extract rooftop counts and areas from satellite imagery of Bo, and to compare these estimates with the rooftop areas collected in our limited ground truth datasets. It may also be easier, particularly if only relatively low-resolution imagery is available, to estimate the aggregate rooftop area rather than to extract the rooftop areas of individual structures.

Correcting for Non-Residential Structures. As discussed previously, if data are not available for non-residential structures, it may necessary to estimate either Tr or Ar from Ts or As, because only the latter two parameters can be directly estimated from the aerial imagery. In some places it might be possible to estimate the ratio of residential to non-residential structures based on relative locations or other distinguishing features. Very small structures – like ones that are latrine (outhouse) sized – can also be removed from consideration. Census data or published business listings could augment this analysis, if available. As a specific example, Aminipouri [13] identified Tanzanian slum areas, and used rooftop extraction to estimate the total rooftop area, assuming that the majority of dwellings were residential. This approach is likely to be invalid in urban or periurban areas, where buildings may include both family dwellings and non-residential structures, or in rural areas that may have many farm structures per residence.

Figure 6 shows the locations of the residential and non-residential structures in Njai Town and Reservation sections. In both sections, the non-residential structures may include ancillary structures that are on the same property as the residences. Without using auxiliary survey data, it would be difficult to distinguish between the two commingled classes of structures from imagery alone. The mean rooftop area for structures in Njai Town is significantly less than the mean rooftop area for the residential structures alone. The mean rooftop area in Njai Town was 172 m^2 ($\sigma = 67\ m^2$) and the mean non-residential rooftop area was 108 m^2 ($\sigma = 71\ m^2$). In Reservation, these figures were 255 m^2 ($\sigma = 126\ m^2$) and 161m^2 ($\sigma = 92\ m^2$), respectively. These distributions are consistent with our observations that the non-residential structures were often small sheds or outdoor cooking facilities. However, some outbuildings are "residence" sized, so it is not possible to discriminate between residential and non-residential buildings based on rooftop area alone.

The difficulty of estimating the ratio of residential to non-residential buildings or rooftop areas is non-trivial. Based on our analysis of the Njai Town and Reservation datasets, this cannot be accomplished based on simple image analysis, or based on the

statistical differences between the rooftop areas of the two groups. One strategy is to exploit expert local knowledge with respect to the distribution of residential and non-residential structures. In this study, having identified all of the residential buildings surveyed in spatially-guided population survey, we were able to estimate both the number (*Ts-Tr*) and total rooftop area (*As-Ar*) of the remaining non-residential buildings in Njai Town and Reservation in the aerial imagery. Participatory GIS (PGIS) is an incredibly valuable tool for the identification of non-residential structures such as markets, businesses, schools, hospitals, nonprofit organizations, and government buildings. Optimally, PGIS can be used in conjunction with analyses of vegetation and other GIS layers predict and validate residential and non-residential zones in other urban and periurban contexts.

Parameter Estimation for Epidemiological Modeling. Given the range and diversity of epidemiological models, it is not possible to determine what minimal sample size is sufficient for all applications. What the present approach enables is the estimated population size as a function of *n*, with confidence intervals of a desired range. For a cross-sectional or cohort study this set of denominator parameters may be sufficient for study design purposes. Each population estimate is the expected value of 2,000 population bootstrap replicates (see Figure 3). (In practice, 200 bootstrap replications are generally considered to be sufficient, but generating 2,000 replicates guarantees the stability of the upper and lower 2.5 percentiles used to estimate the limits of the 0.95 CI [17].) The 2,000 population bootstrap estimates can also be saved for each sample size *n* (Figure 3, step 4a). This provides a number of advantages: Alternative measures of uncertainty can be calculated for each sample size distribution as required, rather than using the percentile confidence intervals. The cumulative distribution function can also be calculated if required from any of the distributions for a given size sample *n* (Figure 3, step 4a). The set of population bootstrap replicate distributions can then be sampled repeatedly to generate the sequence of Monte Carlo inputs required for *Population-Based Microsimulation* (PMS) models (also known as agent-based models), an approach recommended by Sharif et al. [24].

An additional consideration is that the choice of the initial random number seeds will also influence the distribution of the parameters generated in the empirical space [25]. This is demonstrated in Figure 4. Changing the random seed had little impact on the uncertainty, but did cause modest variations in the expected value of the population bootstrap for a given sample size *n*. For PMS models, it is probably best practice to generate and sample multiple distributions of the population bootstrap estimation for a sample of size *n*, each with 2,000 replicates and a different initial random number seed. To achieve even sampling throughout a complex parameter space, Sharif suggests using a Latin hypercube sampling algorithm [24].

Conclusions

Several studies have demonstrated spatial population density estimation using only satellite sensor data [26], acknowledging the limitations of those approaches and recommending combining household data with high resolution remote sensing information [27]. In 2009, no complete municipal maps existed for Bo, Sierra Leone, despite the existence of 68 recognized sections, the boundaries of which were well-known to local populations. In order to establish spatial epidemiology capabilities in Bo, we took an approach from the onset that combines satellite imagery and GIS with local knowledge [14]. The data used in this study came from a larger field survey aimed at obtaining community health data from the 20 municipal sections described in this paper [28–30]. Given the large ground truth dataset afforded by the field survey, we simulated the collection of survey data using samples of varying sizes and a bootstrap population algorithm. Occupancy-based and rooftop-area-per-person-based total population estimators converged correctly on the measured population as the simulated sample size *n* approached the size *N* of the dataset. The uncertainty estimates may be useful designing a resampling study to update the population estimate, while reducing the size of the total number of samples to be collected. These specific findings cannot not be extrapolated to a different set of sections without the making the strong assumption that the new area is demographically comparable with the original survey area, in the sense of having the same underlying distributions of occupants/residential structure. Another advantage of the population bootstrap is that the distributions of the bootstrap population estimates can be saved for each sample of size *n*, and subsequently used to estimate alternative estimates of uncertainty.

The choice of which estimator to use, occupancy based or rooftop area based, will depend upon factors discussed earlier, including (1) the apparent reduction in uncertainty achieved by the occupancy-based estimator to the rooftop area-based estimator; (2) the relative difficulty of estimating the required scale factors (*Ar*, *Tr*, *As*, or *Ts*); and (3) the composition of the survey data.

If only residential buildings are surveyed, as might be dictated by schedule or financing, either *Ar* or *Tr* must be estimated. In this simulation study, both of the residential scale factors, *Ar* and *Tr*, were known for datasets *DS02*, *DS03*, and *DS04*. If both residential and non-residential buildings are surveyed, and represented proportionally in each simulated survey of size *n*, only *As* or *Ts* are required as scale factors. *As* and *Ts* can potentially be inferred directly from aerial imagery of the total area, because it is not necessary to distinguish between residential and non-residential structures.

In this study, we have shown that it is possible to ascertain the appropriate sampling and scale factors by combining local knowledge, satellite imagery, GIS, and surveys of a limited number of households in order to produce robust estimates of total populations. For the type of resource-challenged environment represented in this study, the process consists of the following steps:

1. Visual examination of satellite imagery by persons having local knowledge and removal from consideration of any large non-residential areas (such as commercial or governmental buildings) in the GIS.
2. GIS-directed household surveying of a portion of the residences in a section needed to achieve a desired confidence interval for population estimates.
3. Calculation of the total number of residential structures *Tr*, or the total residential rooftop area *Ar*, to deduce appropriate scale factors (Table 3). Alternatively, if the survey spatially encoded both residential and non-residential structures, the total number of structures *Ts* and/or the total rooftop area *As* provide the scale factors.
4. Use of the scale factors, along with datasets of household surveys, to estimate the population using population bootstrap statistical methods, including the uncertainty in the population estimate as a function of simulated sample size.

We anticipate that refinements of the approach specific to a geographical area will be necessary when extrapolating from an area comprised almost exclusively of single-level residential structures to an area with multiple level ones, as well as those having other unique characteristics. Spatial distributions of population strata, while not estimated here, are a logical extension

of total population estimates, and are made possible by defining such strata in the household survey step.

Another potential application of this approach is for augmenting population estimates derived from satellite imagery alone. In [26], Harvey develops regression models for population estimation that aggregate several different remote sensing indicators, but do not use survey data or rooftop extractions directly. The population space is tested against Australian census data. As noted in [26]:

> There was also evidence of estimation bias associated with the somewhat higher population densities in the secondary study area. *This seems to be related to smaller lot sizes and a higher spatial concentration of separate houses, rather than to substantial differences in the distribution of other types of residential structure*.... It is concluded that the potential of this methodology is limited by heterogeneity of both land cover and population density within the individual CDs [collection districts], and that further improvements are in principle unlikely using this approach. *In particular, the sacrifice of detailed spatial information leaves no way to respond to the problem of over-estimation of population in large areas of low density*. (page 2093, italics added)

The first comment emphasizes the importance of collecting local survey data. Although we have not addressed housing density, given the number of resident structures per section and the areas of each section, the density of residential housing can be readily calculated. The second comment is especially applicable to areas like Reservation, where there are a large number of commercial and government buildings and relatively few residential structures. If the residential and non-residential structures can be differentiated in the imagery, or resolved by surveying the area, the scale factors for both population estimators can be estimated. In summary, it appears that some of the biases in population estimates based on satellite imagery alone could be addressed by analyzing supplementary survey data and/or structural-level imagery.

The ability to quickly estimate total population size with reasonable precision based on satellite or aerial imagery, digitization of rooftops, and demographic surveys of a limited sample of households has important applications for epidemiology and public health, the social sciences, and emergency response. For example, in disaster areas the methods described in this paper could be used to identify permanent and temporary shelters from aerial photographs, guide enumerators to a randomly sampled subset of dwellings, and use those household survey results to estimate the total number of affected persons and to identify resource needs. This critical and timely information about affected communities could contribute to improved delivery of goods and services and facilitate better coordination among response agencies, and might ultimately lead to saved lives. Widespread adoption of these techniques in support of relief activities, community development, and social research will not be possible without improved (and less expensive) digitization software, but the field tests we conducted in Sierra Leone suggest that a process of mapping, sampling, surveying, and bootstrapping can be successfully implemented in low-resource settings to generate accurate population estimates.

Acknowledgments

Disclaimer

The views expressed herein are those of the individual authors and do not reflect views of the Department of the Navy or the Department of Defense.

Author Contributions

Conceived and designed the experiments: RH. Performed the experiments: RH. Analyzed the data: RH JDA. Contributed reagents/materials/analysis tools: RA ASB UB JML KHJ APM DAS. Wrote the paper: RH JDA RA KHJ DAS.

References

1. Viel JF, Tran A (2009) Estimating denominators satellite-based population estimates at a fine spatial resolution in a European urban area. Epidemiology 20: 214–222.
2. Gomez-Elipe A, Otero A, van Herp M, Aguirre-Jaime A (2007) Forecasting malaria incidence based on monthly case reports and environmental factors in Karuzi, Burundi, 1997–2003. Malar J 6: 129.
3. Hethcote HW (2000) The mathematics of infectious diseases. SIAM Rev Soc Ind Appl Math 42: 599–653.
4. Katriel G, Yaari R, Huppert A, Roll U, Stone L (2011) Modelling the initial phase of an epidemic using incidence and infection network data: 2009 H1N1 pandemic in Israel as a case study. J R Soc Interface 8: 856–867.
5. White LF, Pagano M (2008) A likelihood-based method for real-time estimation of the serial interval and reproductive number of an epidemic. Stat Med 27: 2999–3016.
6. Archer B, Tempia S, White L, Pagano M, Cohen C (2012) Reproductive number and serial interval of the first wave of influenza A(H1N1)pdm09 virus in South Africa. PLoS One 7: e49482.
7. Wu S, Qiu X, Wan (2005) Population estimation methods in GIS and remote sensing: A review. GIScience Remote Sensing 45: 80–96.
8. Bhaduri B, Bright E, Coleman P, Urban M (2007) Landscan USA: a high-resolution geospatial and temporal modeling approach for population distribution and dynamics. GeoJournal 69: 103–117.
9. Balk D, Deichmann U, Yetman G, Pozzi F, Hay S, et al. (2006) Determining global population distribution: Methods, applications and data. Adv Parasitol 62: 119–156.
10. Hay SI, Guerra C, Gething P, Patil A, Tatem A, et al. (2009) A world malaria map: *Plasmodium falciparum* endemicity in 2007. PLoS Med 6: e1000048.
11. Magalhães RJS, Clements ACA, Patil AP, Gething PW, Brooker S (2011) The applications of model-based geostatistics in helminth epidemiology and control. Adv Parasitol 74: 267–296.
12. Checchi F, Stewart B, Palmer J, Grundy C (2013) Validity and feasibility of a satellite imagery-based method for rapid estimation of displaced populations. Int J Health Geogr 12: 12.
13. Aminipouri M, Sliuzas R, Kuffer M (2009) Object-oriented analysis of very high resolution orthophotos for estimating the population of slum areas, case of Dar-Es-Salaam, Tanzania. ISPRS XXXVII-1-4-7/W5.
14. Ansumana R, Malanoski AP, Bockarie AS, Sundufu AJ, Jimmy DH, et al. (2010) Enabling methods for community health mapping in developing countries. Int J Health Geogr 9: 56.
15. Davison AC, Hinkley DV (1997) Bootstrap Methods and Their Application. Cambridge University Press, 582 pp.
16. Ripley B, Canty A (2013) boot: Bootstrap R (S-Plus) Functions - R package, 1.3-9 edition. Available: http://cran.r-project.org/web/packages/boot/index.html. Accessed 2013 October 11.
17. Efron B, Tibshirani RJ (1993) An Introduction to the Bootstrap. New York: Chapman & Hall, 436 pp.
18. Wildman J, Hollingsworth B (2002) On generating confidence intervals for nonparametric methods.
19. Stine R (1989) An introduction to bootstrap methods: Examples and ideas. Sociol Method Res 18: 243–291.
20. Collins R, Hanson A, Riseman E, Schultz H (1995) Automatic extraction of buildings and terrain from aerial images. Ascona, Switzerland: Proc. Ascona Workshop, Automatic Extraction of Man-Made Objects from Aerial and Space Images, pp. 169–178.
21. Xiao Y, Lim SK, Tan TS, Tay SC (2004) Feature extraction using very high resolution satellite imagery. In: Geoscience and Remote Sensing Symposium, 2004. IGARSS '04. Proceedings. 2004 IEEE International.
22. Blaschke T (2010) Object based image analysis for remote sensing (review article). ISPRS J Photogramm Remote Sens 65: 2–16.
23. Wong TH, Mansor S, Mispan M, Ahmad N, Sulaiman W (2003) Feature extraction based on object-oriented analysis. In: Proceedings of ATC 2003 Conference.
24. Sharif B, Kopec JA, Wong H, Finès P, Sayre EC, et al. (2012) Uncertainty analysis in population-based disease microsimulation models. Epidemiol Res Int 2012: 14.

25. Wolf DA (2001) The role of microsimulation in longitudinal data analysis. Can Stud Popul 28: 313–339.
26. Harvey JT (2002) Estimating census district populations from satellite imagery: Some approaches and limitations. Int J Remote Sens 23: 2071–2095.
27. Wu C, Murray AT (2007) Population estimation using landsat enhanced thematic mapper imagery. Geogr Anal 39: 26–43.
28. Jacobsen KH, Abdirahman HA, Ansumana R, Bockarie AS, Bangura U, et al. (2012) Home birth and hospital birth trends in Bo, Sierra Leone. Acta Obstet Gynecol Scand 91(6): 750–753.
29. Jacobsen KH, Ansumana R, Abdirahman HA, Bockarie AS, Bangura U, et al. (2012) Considerations in the selection of healthcare providers for mothers and children in Bo, Sierra Leone: Reputation, cost, and location. Int Health 4(4): 307–313.
30. Ansumana R, Jacobsen KH, Gbakima AA, Hodges MH, Lamin JM, et al. (2013) Presumptive self-diagnosis of malaria and other febrile illnesses in Sierra Leone. Pan Afr Med J 15: 34.
